# Cost-Effective 3D Printing of Geopolymer Concrete Enhanced by Na-CMC as a Viscosity Modifier

**DOI:** 10.1021/acsomega.6c03385

**Published:** 2026-07-20

**Authors:** Siim Koor, Hans Priks, Tarmo Tamm

**Affiliations:** Institute of Technology, 37546 University of Tartu, 1 Nooruse St., Tartu 50411, Estonia

## Abstract

This study presents an experimental investigation into the development of a broadly accessible, research-scale geopolymer concrete (GPC) 3D printing platform and formulation. Concrete 3D printing is a rapidly emerging production method that remains underutilized, largely due to legal and regulatory restrictions and, to a degree, the high barrier to entry in the associated research stemming from expensive specialized hardware. In this work, a low-cost tabletop clay printer was modified for GPC production, demonstrating that a dedicated geopolymer formulation can be effectively utilized for additive manufacturing as a low-carbon alternative to conventional concrete. The mix design was engineered to favor geopolymerization while maintaining printability, employing sodium carboxymethyl cellulose (Na-CMC) as a viscosity-modifying additive to enable a low water-to-solid ratio while maintaining workability. The influence of formulation and processing on extrusion behavior and defect formation is discussed. The printer modification protocol and recipe mix design are described in detail to ensure reproducibility and interstudy comparability. The resulting material demonstrated successful polymerization and solid mechanical performance, confirmed by 7-day direct tensile testing of 3D-printed dog-bone specimens (1.1 ± 0.2 MPa), multiscale defect analysis, and attenuated total reflectance Fourier transform infrared (ATR-FTIR) spectroscopy.

## Graphical abstract

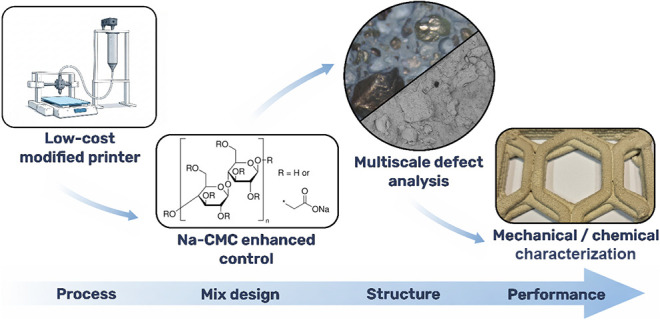

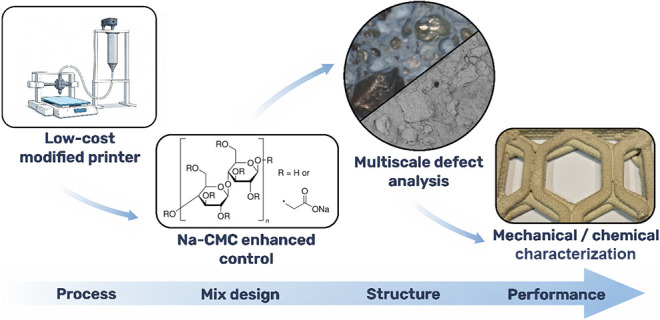

## 1 Introduction

The use of geopolymers and their application as a binder in geopolymer concrete (GPC) in construction is an idea that emerged essentially alongside J. Davidovits’ first conceptualization of clay-like mineral polymers as “geopolymers” in 1979.^1^ However, only recently have these materials started to reach prototype applications in the construction sector, largely hampered by Portland-cement-centered legislation (especially in the European Union) and a lack of fundamental understanding of GPC among sector experts. Despite these restraining circumstances, increasingly intense environmental measures in effect in several world regions, forcing the construction sector to search for alternatives, have allowed GPC to offer competition to more conventional materials. Most notably, 3–4 Gt of ordinary Portland cement (OPC) is produced every year, which is estimated to result in 1.5–3 Gt of CO_2_ emitted, largely as a direct chemical product of limestone calcination, accounting for approximately 7–8% of global anthropogenic CO_2_ emissions per annum.^2,3^ It should be noted that these widely cited statistics use well-established but dated methodology, cemented in the early 2000s by van Oss and Padovani, applied to modern production data.^4,5^ Nevertheless, unlike OPC clinker production, geopolymerization does not directly generate CO_2_ through carbonate decomposition. However, as with OPC-based materials, the environmental benefit of GPC depends on secondary and embodied emissions associated with precursor production, alkaline reagents, admixtures such as Na-CMC, heating, transport, and other processing steps. Depending on the specific formulation and life-cycle boundaries considered, GPC has been estimated to reduce CO_2_ emissions by 40–80% compared with OPC-based materials, making it a potentially attractive lower-carbon alternative.^6,7^

From a formulation perspective, GPC performance is strongly governed by precursor chemistry, alkaline reagent composition, and water content. Previous studies using analytical techniques such as FTIR and NMR have shown that effective Si/Al ratios are generally reported between 1.5 and 2,^8–10^ while Al/Na ratios between 0.8 and 1.25 are commonly associated with suitable charge balance between sodium and tetrahedral aluminum in the geopolymer network.^8,11^ Similarly, H_2_O/Na_2_O ratios around 10 ± 2 are often used to provide sufficient ionic mobility while limiting excess unreacted alkali or carbonate formation.^10,12^ In practical mix design terms, these considerations are commonly associated with W/S ratios of approximately 0.4–0.5,^12,13^ although printable formulations may require lower water content to achieve dimensional stability after extrusion.

Several GPC production methods have been proposed and, to a degree, utilized, for example, preplaced aggregate casting^14^ and spray deposition.^15^ One of the (arguably) novel methods is additive manufacturing or 3D printing. The benefits of concrete 3D printing are easily apparent—reduced labor demand, increased material efficiency, and the availability of more advanced geometries, to name a few. However, its drawbacks are equally evident, among which is the lack of standards and regulatory frameworks for additive manufacturing in the construction sector (in addition to issues concerning GPC legislation as a material).^16,17^ While ISO/ASTM 52939:2023 ^18^ provides foundational qualification and quality assurance principles for construction material additive manufacturing, it explicitly does not specify any material test methods or structural designs. Much of this lack of standardization can be attributed to limited practical familiarity among industry stakeholders, as GPC and concrete 3D printing both require a technically demanding combination of material design, process control, and performance validation. Broader reviews of GPC implementation identify key barriers, including supply chain limitations, lack of performance-based standards, and curing sensitivity.^19,20^ Though direct academic evidence quantifying sector familiarity remains scarce, a study concerning the Kuwait construction industry reports that only approximately 20% of experts have high familiarity with concrete 3D printing.^21^ This lack of awareness, in turn, could stem from the fact that concrete (including GPC) 3D printing entails a technically demanding combination of hardware calibration, mix design, and rheology control, which creates a high barrier to entry, as the performance of extrusion-based systems is strongly governed by the interaction between material properties, extrusion system design, and process control.^22,23^ As a result, it is not often used in teaching, demonstrations, and preliminary research—therefore, only people with in-depth know-how tend to engage with the fundamentals.^24,25^ Accordingly, the present work addresses the need for an accessible research-scale platform for formulation development, printability assessment, and early-stage process understanding, rather than a construction-scale printing system.

The aforementioned high barrier to entry can mostly be attributed to the high initial cost of small-scale, research-level commercial hardware. Several tabletop concrete printers suitable for GPC extrusion are available on the market, however, these systems tend to be accompanied by price points requiring significant financial commitment, often on the order of tens of thousands of dollars. A cost analysis of commercial printers, not explicitly focused on GPC applications, has been compiled by Sovetova and Calautit.^26^ They conclude that small-scale 3D printers suitable for masonry material production cost between USD 4999 to over 23 000. This price range remains a substantial barrier for teaching, demonstration, and early-stage formulation studies, where the aim is often parameter screening rather than construction-scale production. Alternatively, self-assembled printing systems have been reported in the literature, although the construction of such hardware requires notable time investment and know-how.^17,24^ While the cost and self-assembly parameters of concrete printers are not usually discussed together in the scientific literature, there is reason to assume that self-assembly offers a substantially lower price point based on established patterns in broader additive manufacturing.^27–29^

To a limited extent, studies also have discussed 3D printer construction specifically for GPC additive manufacturing, often detailing custom gantry systems, pump–hose layouts, and nozzle assemblies tailored to highly thixotropic mixes.^30–32^ These works, however, predominantly focus on printer design and operation, while deeper materials characterization of printed material often falls outside of their primary scope. Conversely, studies centered on geopolymer material properties treat printer hardware as a predefined or auxiliary component, with validation efforts primarily focused on material formulation and performance.^33,34^ The present work is positioned at this interface by combining detailed low-cost hardware modification, print-parameter calibration, and materials-science validation within a single reproducible research-scale platform.

A prevalent subcategory of self-assembly in GPC research involves the use and modification of commercial tabletop clay printers. Many studies report such applications of printer platforms like PotterBot, Delta WASP, and a few others, however, they typically restrict the discussion of hardware to brief mentions of parameters such as nozzle diameter, extrusion principle, or pump type and rarely provide more extensive detail on the overall printing setup.^35–38^ Only a limited number of studies provide detailed descriptions of printer modifications and print parameter calibration,^39^ as this tends to fall outside their primary research focus. Consequently, the literature exhibits a notable lack of thoroughly documented printer-modification and application guidelines presented together with materials-science analysis and explicit structure–process–property linkages—a gap this work is designed to fill.

In this study, a commercially available and highly affordable clay 3D printer was modified for tabletop-scale concrete extrusion, specifically for concrete that uses geopolymer as the binding matrix, i.e., geopolymer concrete. Modifications to the printer were implemented using widely available hardware components to maximize ease of adoption. The entire hardware rework was systematically documented and is described in a way that ensures complete reproducibility and assumes no prior in-depth technical expertise. Importantly, the system is intended for research-scale formulation development rather than construction-scale production, in practical use, printable material batches can range from approximately 200 g to 1.5 kg, enabling rapid experimental iteration with substantially lower raw material demand than larger laboratory or prototype-scale concrete printing systems. The hardware setup is complemented by a compatible mix design that utilizes additives, such as Na-CMC, to reach sufficient dimensional stability and a high degree of polymerization, enabling continuous strand extrusion and stable layer buildup. Basic correlations between extrusion parameters, fresh-state behavior, and print quality are discussed, illustrating the operational window of the system. By demonstrating that functional GPC printing can be achieved with accessible, low-cost equipment, this study aims to promote wider GPC printing adoption and lower the entry barrier for future research in the field.

## 2 Materials and Methods

### 2.1 Printer Selection and Modification

The printer selected for modification was the Tronxy Moore 2 Pro (Figure 1), an affordable, moving-bed Cartesian system originally designed for home-use tabletop clay printing. It was chosen due to its overall simplicity, including a user interface that requires no prior experience. PrusaSlicer v2.9.0 and Repetier-Host v2.3.2 were selected for model slicing and printer communication, respectively, both of which offer native compatibility with the Tronxy platform. While Repetier–Host is not strictly necessary for operation, it offers convenient live monitoring, calibration, and manual control of the printer.

**Figure 1:**
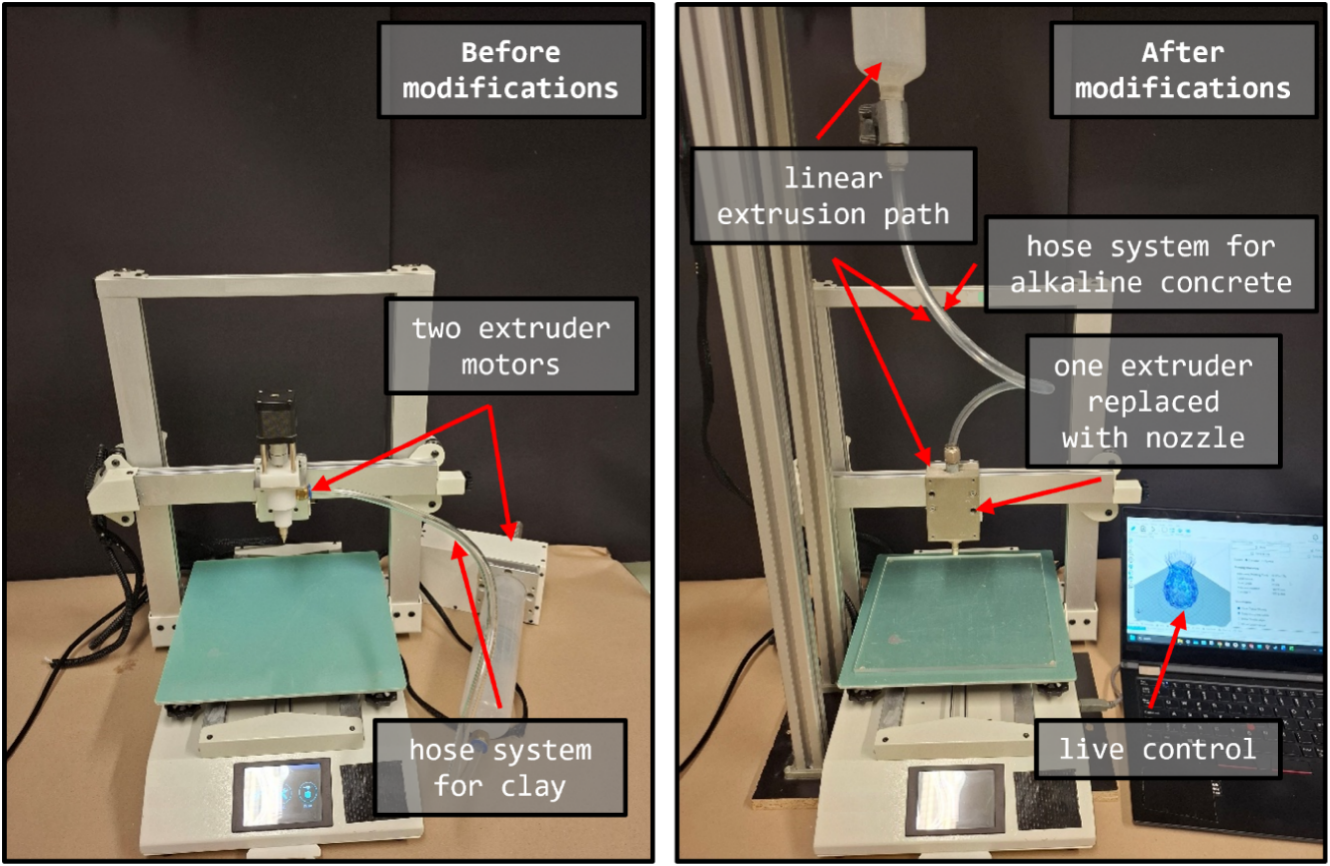
Comparison of the stock Tronxy Moore 2 Pro printer (left) and the modified system reported in this study (right). Highlighted elements indicate the main hardware modifications implemented for GPC extrusion.

In its default configuration, the printer is not suitable for 3D printing of most concrete and GPC mixes because of low extrusion volume, abrasion-related hardware degradation, and insufficient alkalinity resistance. The system was therefore modified for GPC extrusion with a maximum grain size of 1 mm, as illustrated in Figure 1, larger grain sizes may be possible but were not investigated in this work. The complete modification protocol and a comprehensive bill of materials with itemized costs are provided in the Supporting Information—sections 2 and 3. The combined cost of the base printer and all modification components remained below 600 €.

### 2.2 Raw Materials

Two main aluminosilicate sources were used—Metamax metakaolin, with a claimed 97.5% purity (SiO_2_ + Al_2_O_3_) and DPT-Soft diatomaceous earth, claimed purity 90% (SiO_2_ + Al_2_O_3_). Additionally, hydrophilic fumed silica Aerosil FS 200 was used to balance relevant ionic ratios. Alkaline reagents were 10 M NaOH solution, prepared from Lach-ner produced pellets, and construction-grade sodium silicate from Kemet RV. Sand used as filler was sourced from a local quarry at Männiku (glaciofluvial delta deposit) and filtered through 1 mm sieve. Sodium carboxymethyl cellulose (Na-CMC), used as viscosity-modifying additive, was produced by CP Kelco (Mw ≈ 30 000 g·mol^–1^). Chemcial composition of raw materials can be found in Supporting Information—section 7.

### 2.3 Mix Parameters

To study the mechanical strength of 3D-printed GPCs, a mix was developed containing approximately 50% filler/aggregate. The primary objective of the formulation was not only to achieve sufficient strength but also to ensure compatibility between the material and the modified printer, i.e., stable extrusion and adequate interlayer adhesion under the selected printing conditions. The raw materials acted either as precursors for the geopolymer binder or as viscosity control agents. The full composition of the 3D printing mix, the mixing order, and the conditions are presented in Figure 2. In addition, two control formulations were prepared as mold-cast specimens: one designed as a high-reactivity metakaolin-based geopolymer mix, selected within composition ranges reported to yield high strength and dense matrices in metakaolin GPC,^8^ and another representing a low-polymerization, gel-like aluminosilicate network.

**Figure 2:**
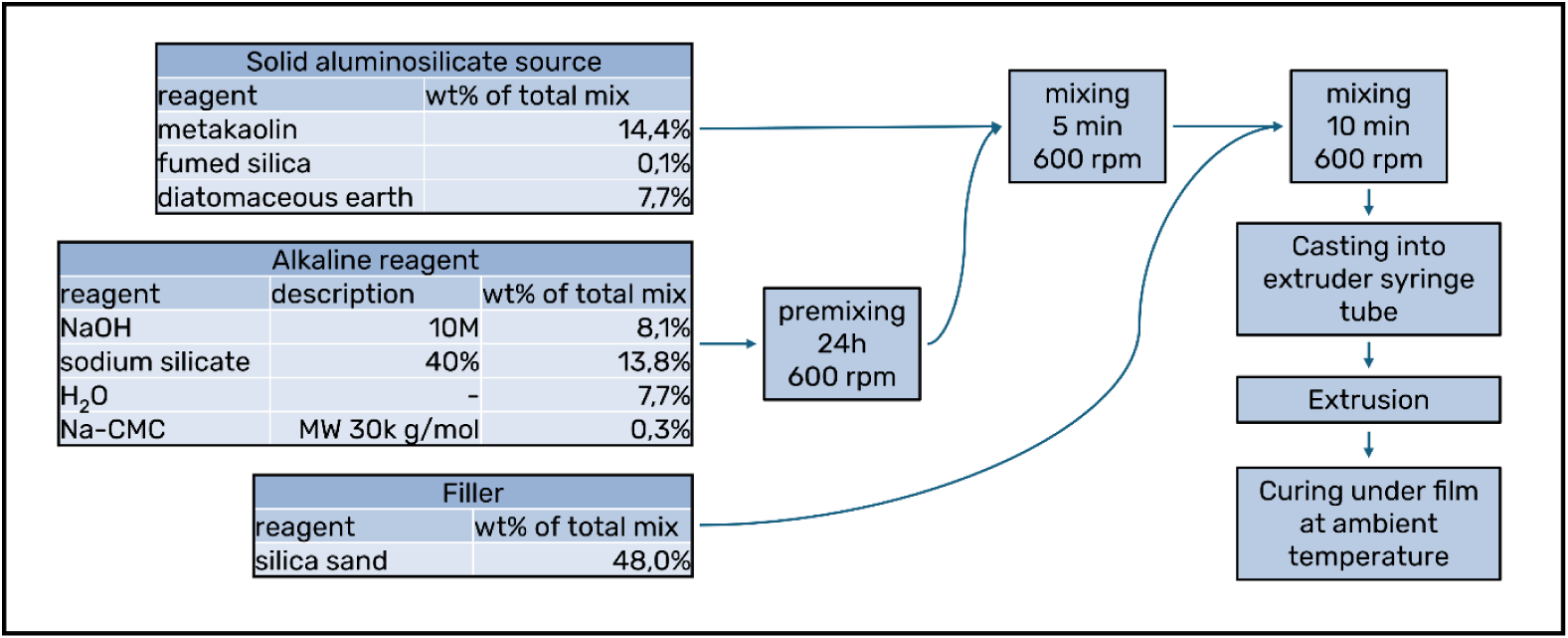
Schematic of the 3D-printing mix design shows the relative mass fractions of each precursor group and the sequential premixing, mixing, and curing steps used before extrusion.

Mix design parameters for mechanical testing samples are listed in Table 1. The target Si/Al, Al/Na, H_2_O/Na, and water-to-solids ratios were selected to fall within ranges commonly associated with effective geopolymerization and mechanically robust aluminosilicate networks, as reported in previous studies, while maintaining workability suitable for extrusion.

**Table 1:** Target Composition Parameters of the GPC Mix Used for Tensile Testing Specimens, Expressed as Key Ratios^a^

Si/Al	Al/Na	H_2_O/Na	W/S	Aggregate
Molar ratio			Mass ratio	Mass percent
2.31	0.86	7.86	0.28	48.0

a The reported molar ratios were calculated from bulk oxide-equivalentraw-material compositions, converted to elemental molar amounts and summed across the relevant mixture components. raw-material compositions, converted to elemental molar amounts and summed across the relevant mixture components.

### 2.4 Printing Procedure and Parameters

A detailed analysis of the print parameters and their influence is presented in Section 3.1 Machine, print, and recipe parameters. Full slicer configuration files, STL geometries, and other relative information are provided in the Supplementary Repository archived in Zenodo and updated on GitHub (Supporting Information—section 1).

Dog-bone specimens were printed using the modified Tronxy printer in a Cartesian, moving-bed configuration with a nozzle diameter of 5.9 mm, a printing speed of 5 mm/s (using stock motion settings for acceleration and jerk) and a nominal line width of 6.0 ± 0.3 mm (95%, *k* = 2,306). The maximum aggregate size in the mix was limited to Ø1 mm to ensure clog-free extrusion. A concentric infill pattern at 100% infill density was used, with an infill-perimeter overlap of 0%, and no dedicated bottom or top solid layers were implemented to avoid nonstandard nozzle movement paths. The layer height was set to 2.5 mm, the effective strand diameter in the slicer was set to 5.5 mm, and an extrusion volume multiplier of 1.2 was applied for all dog-bone prints.

### 2.5 Material Characterization

Apparent viscosity measurements were performed by using an NDJ-5S rotary viscometer operated at 6 rpm. Compositionally adjusted model mixtures were used to enable a controlled relative comparison between mixtures with and without Na-CMC. Both model mixtures contained 30% more water than the printable formulation, while the Na-CMC-containing mixture used one-third of the Na-CMC dosage used in the printable formulation. In addition, a conical minislump test was performed 15 min after mixing, in accordance with the methodology proposed by Provis et al.,^40^ to assess fresh-state flow and shape retention.

To characterize the mechanical properties of the printed material, 7-day direct tensile strength measurements were performed on 3D-printed dog-bone specimens (cured at 100% RH, 21 ± 1 °C and then dried under vacuum). The dog-bone geometry was intentionally selected to consistently promote fracture in the desired narrower region in the middle part of the specimen, following concepts used for tensile testing of other masonry materials.^41^ The printed specimens were loaded uniaxially in tension until failure at a displacement rate of 1 mm/min (Figure 3). Friction-type grips were used to mount the specimens to avoid microcracking. A total of 12 samples, printed in four different series with identical parameters, were tested, and tensile strength was calculated from all tested specimens with no exclusions. This sample size was considered sufficient for the scope of this study, as the specimens represented repeated print series and showed relatively low scatter. The result is therefore reported as the mean at a 95% confidence interval to account for sample-to-sample variability.

**Figure 3:**
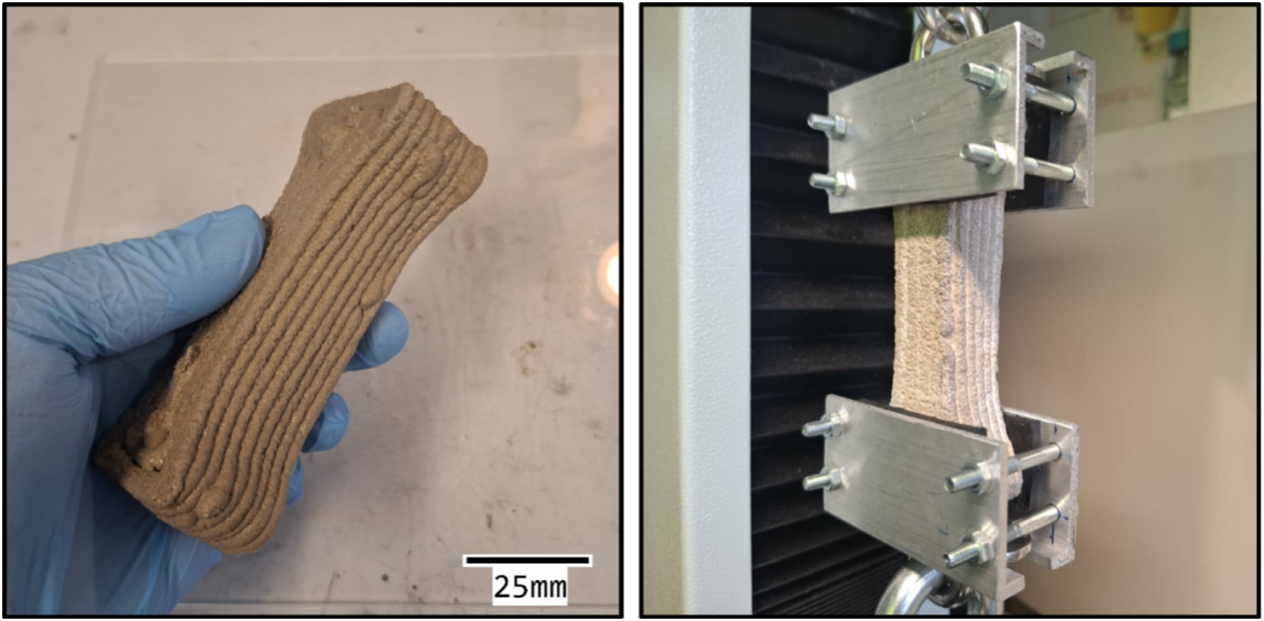
Dog-bone specimen shortly after printing (left) and after 7 days, mounted in the direct tensile testing rig (right). The rig employs friction gripping and provides a single degree of freedom to ensure uniaxial stress at fracture.

Multiscale defect analysis was performed, including scanning electron microscopy (SEM) and energy-dispersive X-ray spectroscopy (EDX). The analysis was performed on fragments taken from tested dog-bone specimens using Hitachi TM-3000 electron microscope operating at 15 kV, equipped with Oxford Instruments SwiftED 3000 EDX analyzer, examining both visually identified defects and representative bulk regions. Additionally, attenuated total reflectance Fourier transform infrared (ATR-FTIR) spectroscopy was performed on Bruker ALPHA I to assess vibrational bands characteristic of geopolymerization reactions in the printed material.

## 3 Results and Discussion

This section combines the results of the experimental study with their interpretation in terms of printer design, process parameters, and material behavior. The influence of machine modifications, print settings, and mix composition on extrusion stability and achievable geometries is described, establishing the operational window of the low-cost GPC printing setup. Mechanical performance, multiscale defects, and ATR-FTIR spectra are analyzed together to relate tensile strength and defect formation to viscosity, print parameters, and the extent of geopolymerization.

### 3.1 Machine, Print, and Recipe Parameters

#### 3.1.1 Machine Design Principles

A conventional clay printer and a fundamental, yet fully operational GPC printer have considerable overlap in operating principles, allowing the former to be utilized as the latter. Nevertheless, modifications were needed to reach a sufficiently optimal performance.

For concrete additive manufacturing, one of the most critical limiting factors regarding the machine parameters is the grain size. While the definition of “concrete” is somewhat ambiguous in the 3D printing context, mixes with maximum grain size of 1 mm, as in this study, could be considered as such. This results in the most fundamental difference from clay printing, where largest particles are less than 0.002 mm in diameter by the definition of ISO 14688–1:2018 ^42^ (Udden-Wentworth scale).

With this in mind, the extrusion system was modified to accommodate larger, more abrasive particles, as illustrated in Figure 1.

Notable modifications included:

- The secondary extruder and its drive motor were removed. In an unmodified configuration, it cannot resist the alkaline slurry and abrasive sand filler and, therefore, cannot maintain consistent material flow between different printing instances.
- The primary syringe extruder motor, material tube, and housing were mounted on a separate free-standing frame above the extruder. This enabled a linear *z*-axis material path with a more rigid hose system and a higher volumetric flow.
- An increase of the hose’s inner diameter from 6.5 mm to 8 mm, using alkali-resistant PVC and stainless steel, effectively doubled the laminar flow rate. The nozzle diameter increased approximately 10x compared to clay printing via interchangeable pneumatic fittings.

With these modifications, printing speeds of 5 mm/s were achieved, fully satisfying the scope of the present study. Overall, the modifications enabled stable extrusion, successful shape retention, and sufficiently high tensile strength of geopolymer concrete with consistent layer deposition and acceptable print resolution, validating the feasibility of adapting clay printing hardware for GPC applications. Owing to the well-balanced viscosity and rheological properties, more advanced shapes can be achieved, such as the dog-bone specimens discussed in this study. Current and adjusted print parameters could also enable more complex print techniques, such as overhangs, bridging to a limited extent, and complex infill patterns, such as illustrated in Figure 5 and Supporting Information—section 4.

#### 3.1.2 Print Parameter Calibration and Control

Nozzle-dependent extrusion volume presets were calibrated to establish a stable baseline. Building upon these presets, fine adjustment of the extrusion volume was achieved through the application of an extrusion volume multiplier, with the most consistent results obtained at a multiplier of 1.2x. This additional adjustment increases the material pressure during extrusion, largely mitigating void formation associated with extrusion lag and geometric underfill.

Print-specific fine-tuning of the extrusion volume was performed through control of the “flow” parameter in the slicer software. The slicer flow parameter was treated as an operationally proportional control variable to volumetric material delivery. Within the investigated range, a linear relationship between flow setting and resulting printed line width was assumed and experimentally evaluated. The flow setting is a precise extrusion volume multiplier that most slicer software packages allow to be conveniently adjusted. As shown in Figure 4, the flow rate exhibits a distinct and approximately linear correlation with the deposited line width within the studied range of 75% to 150%, resulting (relative to the base value of 100%) in an 11% reduction and a 22% increase in line width, respectively. The corresponding linear fit parameters are provided in the Supporting Information—section 3. This wide adjustment range provides an effective means of controlling the deposited layer width in the studied printing setup, enabling more advanced print geometries and offering a practical correction mechanism for common extrusion-related print artifacts.

**Figure 4:**
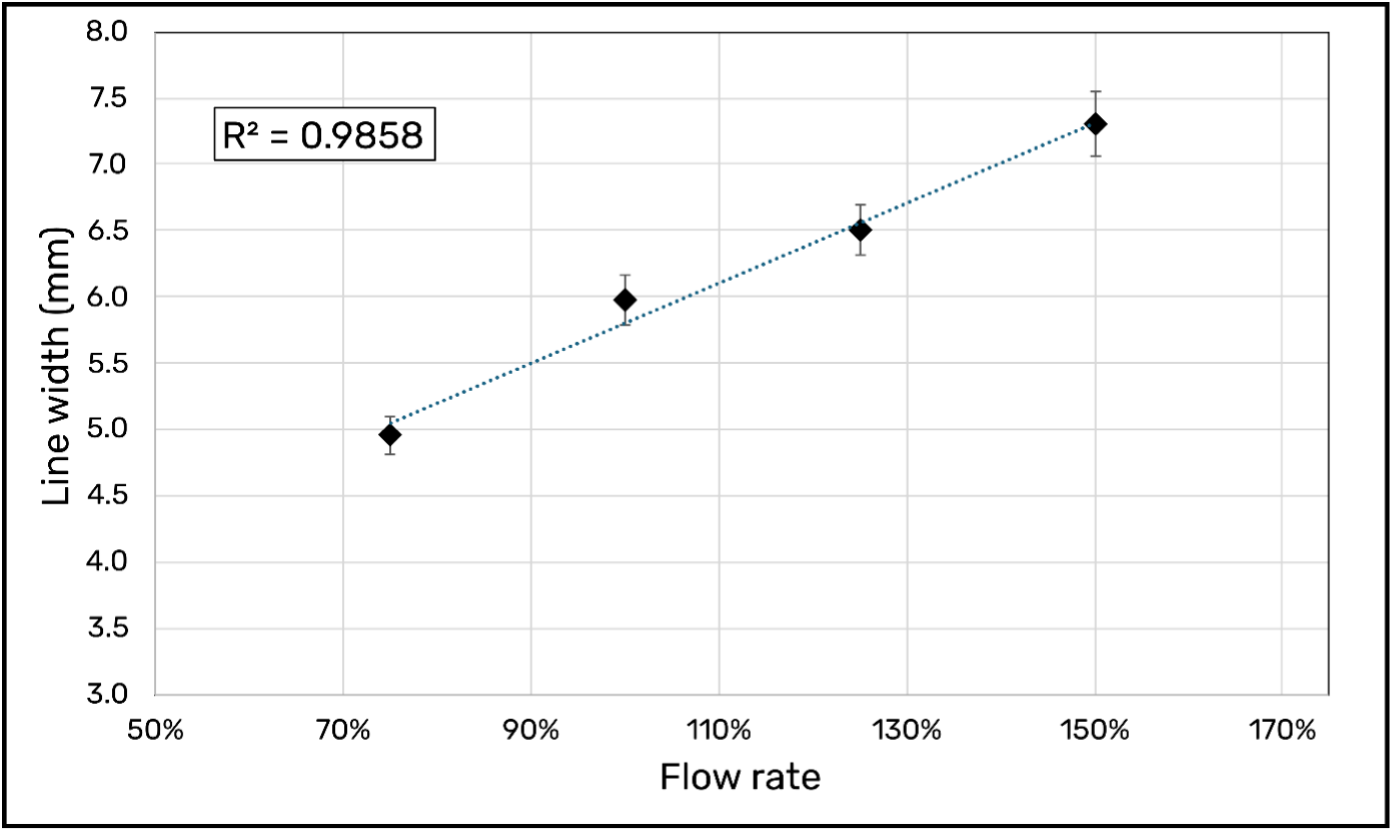
Deposited line width, utilizing 5.9 mm nozzle, as a function of volumetric flow (with 100% corresponding to the default flow rate). The dashed line represents a linear regression.

Layer height (the distance between successive layers along the *z*-axis) was another key parameter calibrated for GPC extrusion. Conventional thermoplastic extrusion typically employs layer heights of 25–80% of the deposited filament diameter. For GPC in this study, however, the operable range proved substantially narrower: layer heights of 30–50% of nozzle diameter provided optimal performance. Most consistent results were achieved with identical layer heights for both initial and subsequent layers, though the mechanistic basis for this observation requires further investigation.

Other print parameters were maintained within conventional ranges (Figure 5). For example:

- Infill density between 20% and 80% in simple patterns such as concentric, rectilinear, and grid. Additionally, in more advanced patterns like honeycombs.
- Outer vertical perimeters numbered between 1 and 5 instances.
- Both nonseamed (spiral vase-style) and seamed (random/aligned) perimeters.
- Print speeds between 1 and 5 mm/s (perimeters, infill, and first/top layers).
- Infill-perimeter overlap was between 5% and 40%.

**Figure 5:**
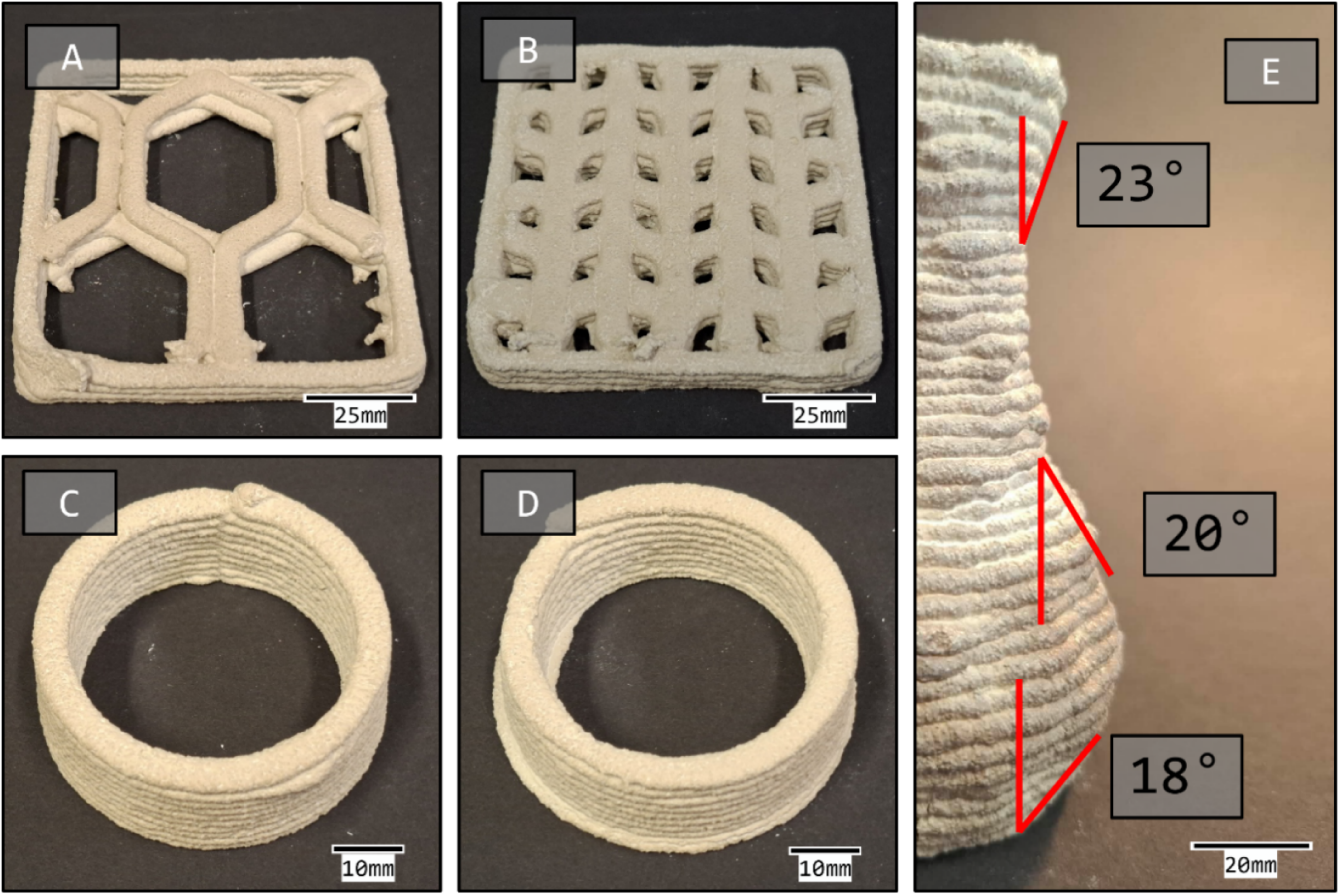
Representative print outcomes used to validate the selected print parameters illustrate the achievable complexity of infill geometries and the general consistency of material deposition. (A) honeycomb infill (25%), (B) grid-like infill (80%), (C) aligned perimeter seams, (D) nonseamed spiral perimeter, and (E) vase geometry print illustrating overhang angles. All values are from set parameters in software, not measured from physical objects. Scale bars are approximate due to perspective effects and nonplanar geometry.

These calibrated parameters enabled stable layer deposition, good interlayer bonding, and reproducible printing geometries.

#### 3.1.3 Print-Compatible Mix Design

The present mix was designed by combining the literature-established geopolymer formulation principles with the workability requirements of the modified printer. The main deviation from commonly reported mix design ranges was the deliberate reduction of the W/S ratio to 0.28. This value was chosen to improve the shape retention of the extruded paste and reduce material slump. Lower water content inherently limits workability and extrusion stability, however, these drawbacks were mitigated by the introduction of sodium carboxymethyl cellulose (Na-CMC) as a viscosity-modifying additive. Na-CMC is an anionic polysaccharide expected to modify fresh-state behavior through water retention, increased viscosity, particle suspension stabilization, and reduced segregation. This adjustment allowed the mixture to retain cohesive flow characteristics while preserving the dimensional stability required for additive layer deposition.

The presented formulation should be understood as one printer-compatible mixture, rather than a universal recipe. However, the modified printer is not inherently limited to this exact composition, and other geopolymer formulations or modified versions of the present recipe are likely to be printable, provided that particle size, abrasiveness, viscosity, cohesion, and setting kinetics remain compatible with the extrusion path and deposition conditions. Indeed, one of the intended uses of the platform is to enable further formulation screening with different precursor combinations, additive dosages, and fine inclusions. In practice, each new formulation would require recalibration of water content, viscosity-modifying additive dosage, extrusion multiplier, print speed, and layer height. Larger or more angular inclusions would be expected to increase the risk of hose/nozzle blockage, underextrusion, and interlayer defects.

### 3.2 Fresh-State Characterization

Fresh-state viscosity characterization was performed using compositionally adjusted model mixtures rather than the final printable formulation. The printable mix was sufficiently stiff and shear-dependent that single-speed apparent viscosity measurements were not expected to provide consistent or directly comparable results for the unmodified and Na-CMC-modified mixtures. Therefore, an adjusted model system was used to characterize the effect of Na-CMC under a deliberately simple and comparable test setup. Both model mixtures contained 30% more water than the printable formulation, and the Na-CMC-containing model mixture used one-third of the Na-CMC dosage used in the printable mix.

Mini-slump testing provided a direct visual indication of the shape-retention effect of Na-CMC (Figure 6). After cone removal, the control mixture showed extensive slump, with the height decreasing from 57 mm to 12 ± 3 mm [95%, *k* = 4.303] and the diameter increasing from 38 mm to 68 ± 1 mm after 1 min. In contrast, the Na-CMC-containing mixture retained substantially greater height, 44 ± 10 mm, and showed only minimal lateral spread, with the diameter increasing from 38 mm to 40 ± 1 mm. These observations confirm that Na-CMC strongly improved dimensional stability in the fresh state.

**Figure 6:**
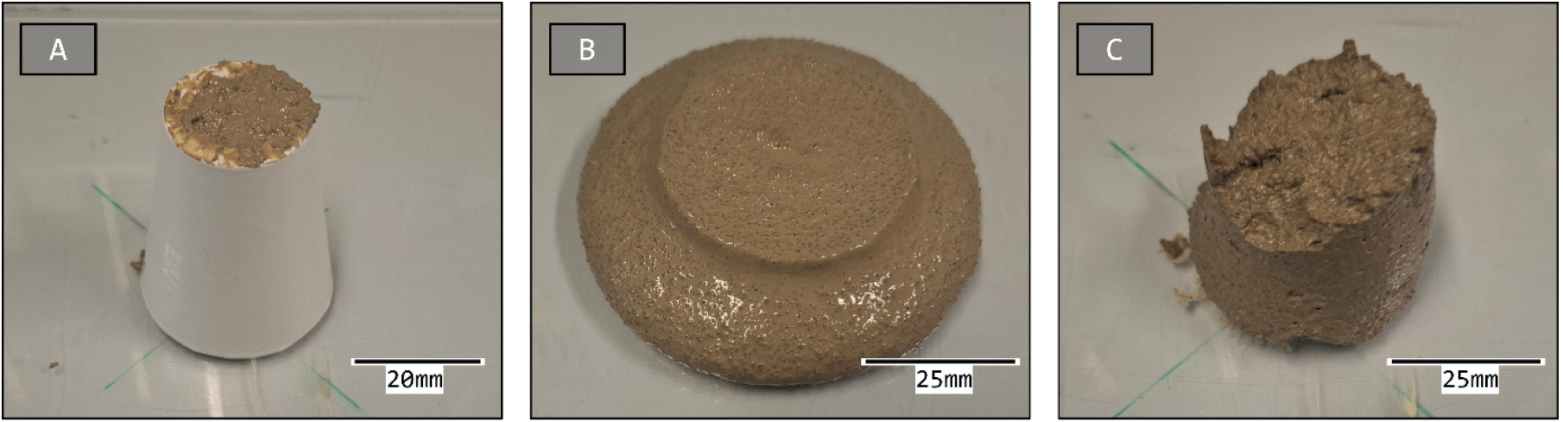
Representative mini-slump test images were used to assess fresh-state shape retention: (A) initial cone geometry before mold removal, (B) control mixture 1 min after mold removal, and (C) Na-CMC-containing mixture 1 min after mold removal. The control mixture exhibited substantial collapse and lateral spread, whereas the Na-CMC-containing mixture retained much of its initial geometry.

Apparent viscosity measurements further confirmed the viscosity-modifying effect of Na-CMC (Figure 7). Throughout the measured period, the Na-CMC-containing mixture exhibited substantially higher apparent viscosity than the control. At 15 min after mixing, the apparent viscosity had reached a relatively stable region, with the control mixture measuring 140 ± 20 mPa·s [95%, *k* = 4.303] and the Na-CMC-containing mixture measuring 560 ± 80 mPa·s. Thus, even with increased water content and reduced Na-CMC dosage relative to the printable formulation, Na-CMC increased the apparent viscosity about four times. Although apparent viscosity gradually decreased for both mixtures during the measurement period, the Na-CMC-containing mixture remained several times more viscous than the control throughout the test. Taken together with the mini-slump results, this indicates that Na-CMC increased both the resistance to flow and the ability of the fresh material to retain its shape. As the model mixtures used increased water content and reduced Na-CMC dosage relative to the printable formulation, the observed differences should be interpreted as conservative evidence of the viscosity-modifying effect of Na-CMC.

**Figure 7:**
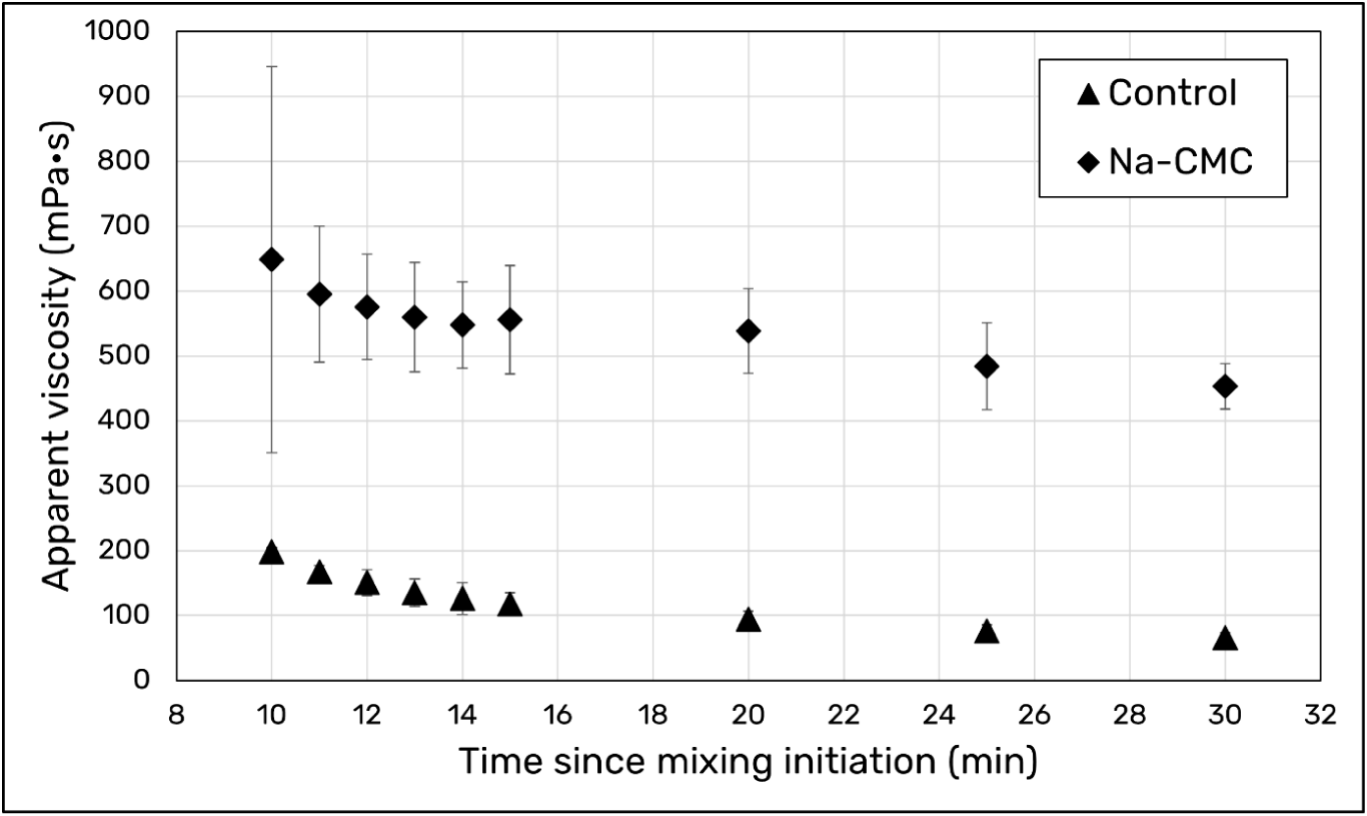
Apparent viscosity of control and Na-CMC-containing model mixtures as a function of time after mixing, measured at 6 rpm. Error bars represent 95% confidence intervals.

### 3.3 Direct Tensile Strength

Average 7-day direct tensile strength of 1.1 ± 0.2 MPa (95%, k = 2.201) was achieved for the printed material. There is a significant amount of literature addressing the tensile strength of geopolymers, however, the majority of reported values are derived from indirect measurements, and the resulting strength is strongly influenced by reinforcing additives, raw material selection, and specimen age. Such indirect measurement values are often reported to be around 5–10% of compressive strength, typically yielding values in the low single-digit MPa range (2–5 MPa).^43,44^ Studies concerning direct measurements also exist, though typically for fiber-reinforced material, placing the values between 0.5 and 4 MPa.^45,46^ A limited number of reports have also focused on the tensile strength of 3D-printed specimens, reporting strength in the range 2–3.5 MPa.^47,48^ These observations indicate that both direct tensile testing and 3D printing can reduce measured tensile capacity compared to mold-cast reference materials.

Furthermore, direct comparison with production-scale concrete printing materials is complicated by differences in printer scale, binder chemistry, aggregate size, reinforcement, curing regime, build direction, specimen geometry, and test method, with most studies reporting compressive or flexural strength rather than direct tensile strength. In this context, the small material volume required by the present tabletop system should be viewed as a practical advantage for early-stage formulation screening, rather than as a limitation of the experimental design. Therefore, despite the limited availability of research regarding direct tensile strength of 3D-printed specimens,^47^ comparable values from similar tensile measurements support the conclusion that the printed material reached a sufficient degree of geopolymer network development, notwithstanding the incorporation of viscosity-modifying additives.

### 3.4 Defect Analysis

It has been well established that the mechanical strength of 3D-printed geopolymer concrete, while competitive, often shows considerably lower values than mold-cast material. However, the evidence from preliminary testing suggests that this reduction can be mostly attributed to defects stemming from workability and printing parameter issues. These studies have resulted in a considerable backlog of reported and qualitatively mapped defects and their likely causes in the scientific literature, though they are rarely documented with the explicit purpose of interstudy comparability.^33,49,50^ Despite this, the study of the defects is an excellent tool for estimating the capability of printers and highlighting the importance of printing parameters and printing mixture properties.

#### 3.4.1 Macroscale

First, it should be noted that defects were present, but not common. The figures below depict exceptionally severe defect cases for illustrative purposes, outlining some defect types (Figure 8). The most persistent defect was the initial layer compression artifact, aka “elephant’s foot” (Figure 8A), a common issue in 3D printing of any material, but especially dominant in materials with with low early-stage stiffness, such as GPC and other masonry materials. The defect can arise from several potential causes. In the present work, it is attributed to the increased pressure in the first layer deposition, since, unlike extruding on top of other printed layers, the rigid print bed does not yield and cannot accommodate pressure with deformation. Furthermore, the relatively slow print speed of 5 mm/s, accompanied by the high extrusion volume inherently required for such large-diameter strand deposition, is likely to cause even higher extrusion pressure. Consequently, the first layer of extruded material spreads in a lateral orientation more than the subsequent layers. In principle, this defect could be mitigated by optimization of extrusion pressure, mainly through the adjustment of layer height or flow ratio of the first layer to subsequent layers. However, due to variations in inter-recipe viscosity, such delicate calibration is likely to be needed for each new recipe instance, which is widely accepted practice in the field. That said, even for a simple system such as the modified Tronxy printer, these calibrations are not inherently difficult to perform, rather, they require certain know-how or persistence.

**Figure 8:**
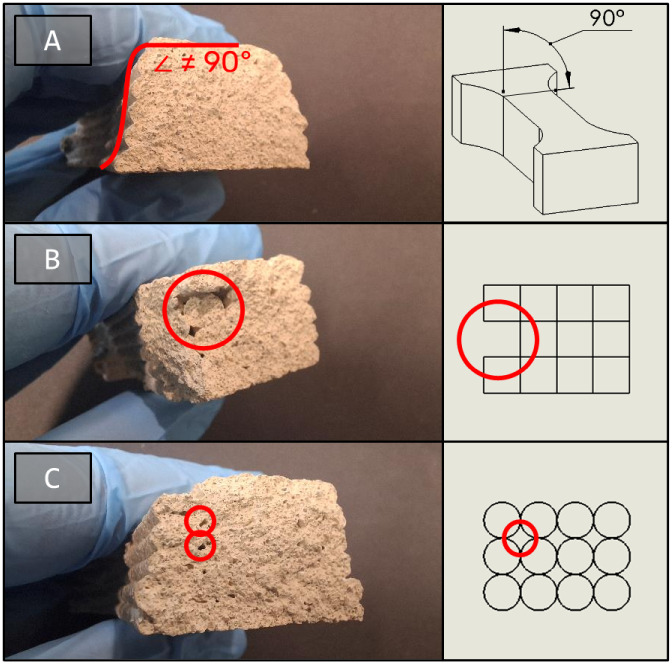
Macroscale defects present in printed materials (left) and explanatory schematics (right): (A) first-layer compression artifact and voids from (B) underextrusion or (C) geometric underfill. Exceptionally severe defect cases were selected for clarity.

Limiting extrusion pressure (including flow rate) too extensively, however, can lead to another notable defect—the presence of voids in the material bulk. These void instances stem either from temporary underextrusion (Figure 8B) or geometric underfill (Figure 8C). Underextrusion is caused by pressure drop, which in turn is caused by inhomogeneity in the extruded slurry, for example, if a local region of the mix has experienced a reduction in free water (e.g., due to segregation or localized dewatering), resulting in a sharp viscosity increase.^51^ This leads to syringe lag in extruder and no material being extruded for a brief period. The lower the actively maintained extrusion pressure, the more severe and common the temporary underextrusion effects can be. The geometric underfill is an issue somewhat more challenging to address. It is caused by using cylinder-shaped extruded paste to fill cuboid-shaped space, leading to voids at the edges of the deposition path. The larger the diameter of the extruded paste, the more severe the geometric inconsistency, and the larger the voids. This can be addressed by intentional over extrusion by increasing the flow rate or reducing nozzle size relative to the target deposited material line width, so the paste expands after deposition to fill the geometric voids. Alternatively, a rectangular nozzle capable of rotation could be employed to better match the deposited geometry. However, such a solution would introduce additional mechanical complexity, which falls outside the scope of this work.

As the examples above demonstrate, extrusion pressure is a delicate yet critical aspect of GPC printing, relying on the optimization of several parameters to achieve the desired material properties and defect tolerance for a given print and application. However, extrusion pressure can be readily controlled (albeit somewhat labor-intensive) through layer height and print speed calibration. On the basis of the described printer setup, the authors estimate that fatal defects occurred in fewer than 5% of printed samples and that this failure rate could be further reduced through recipe-specific print parameter optimization.

These observations highlight that macroscale defects in printed GPCs are primarily governed by extrusion pressure management, rather than intrinsic material limitations.

#### 3.4.2 Meso- and Microscale

Mesoscale mostly describes the interactions between the geopolymer matrix and the sand grain filler. Micrographs illustrating defects present at the mesoscale and the qualitative elemental distribution of Si and Al are shown in Figure 9. The mapping of Si and Al ions shows clear regions with increased Si presence, meanwhile the regions in between have signals from both Si and Al. The former corresponds to the filler material—sand, as silica sand (river source) has, on average, 6.4% and almost always less than 16% of Al incorporated on the surface.^52^ The studied geopolymer matrix, however, has an approximate ionic ratio of Si/Al = 2.3, which explains the mixed signal from the intergrain regions.

**Figure 9:**
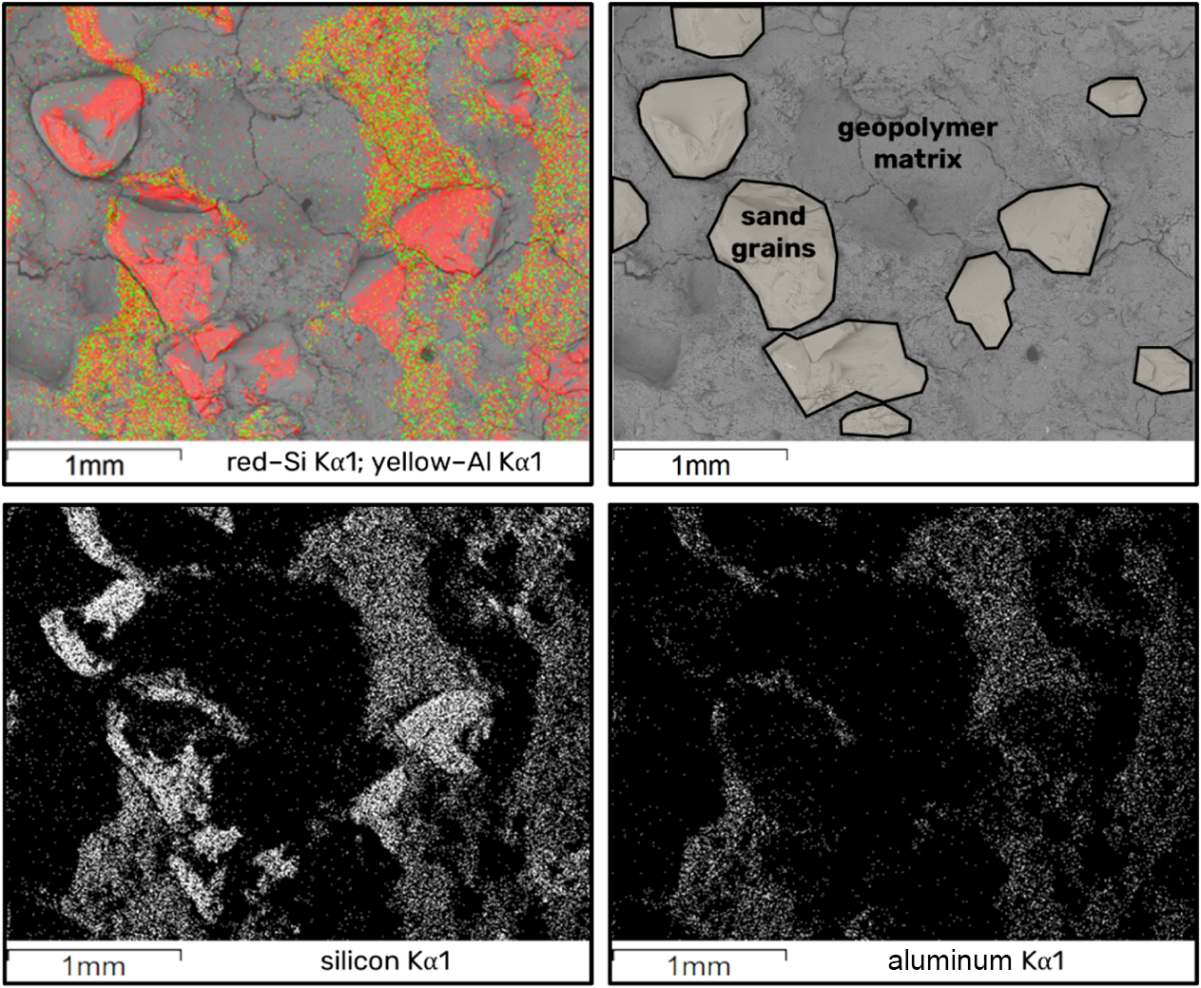
Mesoscale study of the cut GPC surface shows elemental mapping of the geopolymer matrix and filler sand grain regions.

Micrographs illustrate geopolymer matrix microcracking, a prominent defect observed in the produced material. It has been well-documented that GPC microcracking is associated with interfacial transition zones (ITZs) between filler particles and the matrix.^53–56^ The geopolymer matrix shrinks in volume as part of the natural curing process, and the resulting restrained eigenstrains tend to concentrate at material heterogeneities such as ITZs, where local fracture resistance may be lower than in the bulk matrix.^55^ That said, it was also clearly observed that, in many cases, ITZs acted as a crack propagation-arresting regions, fulfilling the intended reinforcing role in the GPC. Furthermore, as the samples studied were those from the tensile tests, at least a part of the microcracking is expected to be due to the tensile strains created during the testing. The issue of microcracking is common in GPC production and could be mitigated by better control of eigenstrains, either through an increase in curing temperature^57^ or adjustment in Si/Al ratio.^58^

Microcracking could additionally be promoted by the relatively low extrusion and general printing speeds. Potentially, this could cause internal stresses around the ITZs due to the filler material being exposed to extrusion stresses for longer and having more time to settle in the deposited layer, leaving behind voids at the ITZs. Furthermore, long print times could cause early curing in suboptimal conditions for the layers deposited first, as the early stages of polymerization initiate within tens of minutes.^59,60^ Therefore, even though the described setup ensures sufficient layer adhesion and near-complete infill at the designed scale (excluding isolated macroscale defects), microcracking could potentially be reduced by the increase in print speed.

### 3.5 Infrared Spectroscopic Verification of Geopolymer Formation

Figure 10 shows the measured ATR-FTIR absorption spectrum for a sample used in the tensile testing, together with high- and low-polymerization control mixes, providing clear evidence of geopolymer aluminosilicate network development in the test samples. Of special interest is the envelope of Si–O–T (T = Si/Al) vibrations encompassing the peaks at 1078 cm^–1^ and 1019 cm^–1^. The former is indicative of Si–O–Si asymmetric mode, one of the most commonly reported bands present in geopolymers. Given the high Si/Al ratio of the recipe, the significant presence of such band is wholly expected. However, such vibrations are also common in metakaolin—one of the main precursors.^61^ Therefore, the 1078 cm^–1^ band, while supportive evidence, is not a determinative identifier of polymerization. The 1019 cm^–1^ absorption, however, is commonly attributed to the asymmetric stretching of T–O–T linkages involving Al in tetrahedral coordination (Si–O–Al) and is widely used as an FTIR indicator of geopolymer gel formation. Occasionally, such environments can also be present in metakaolin, but there they are considered a secondary or tertiary configuration, resulting in a much weaker signal.^62^

**Figure 10:**
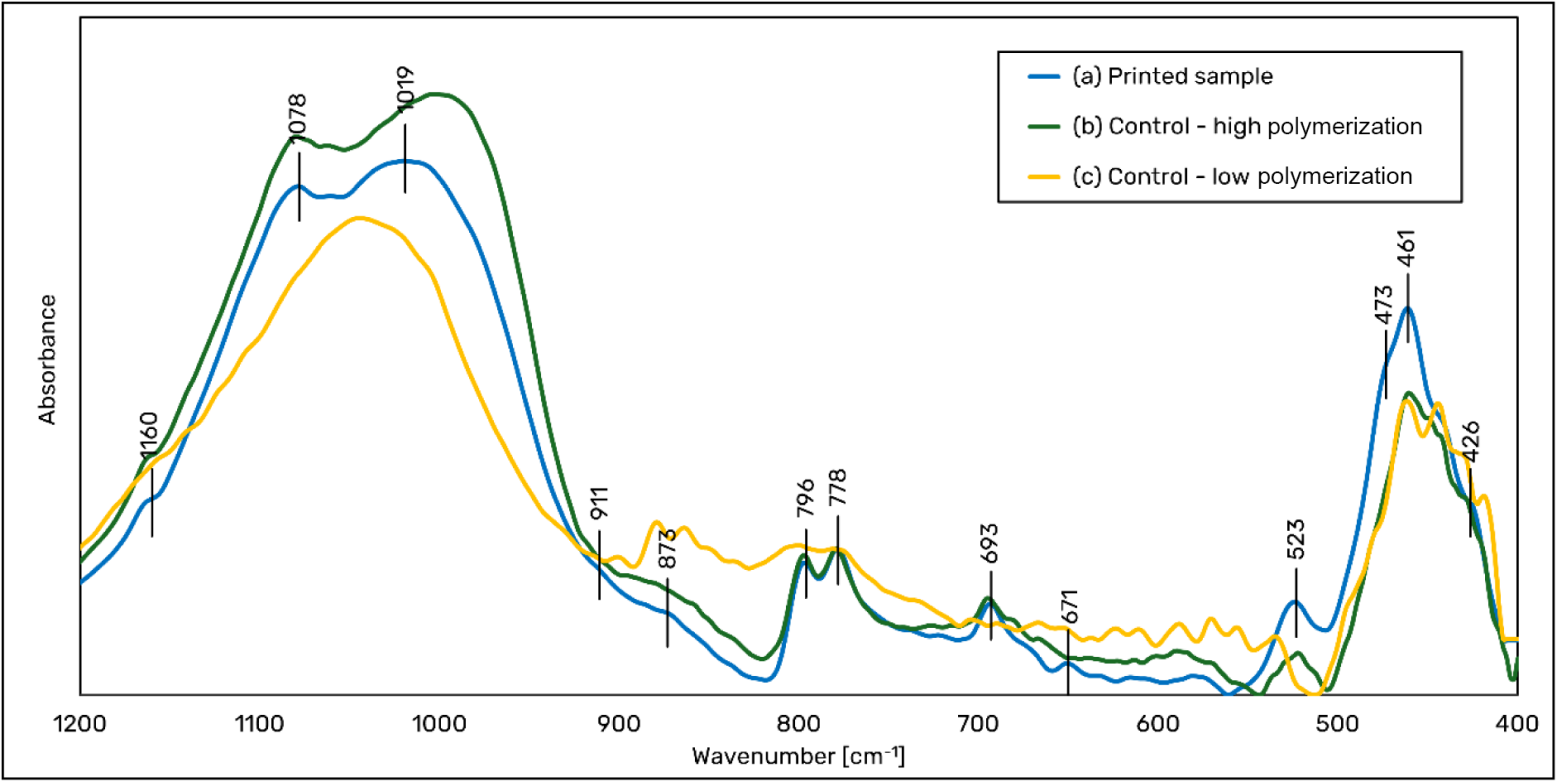
ATR-FTIR spectra of (a) a printed dog-bone specimen used for tensile testing, (b) a mold-cast control sample exhibiting a high degree of geopolymerization, and (c) a mold-cast control sample with a low degree of geopolymerization. Normalized for area in the range of 1200 cm^–1^ to 400 cm^–1^ to facilitate comparison of aluminosilicate network features.

Peaks at 796 cm^–1^ and 778 cm^–1^ align well with the signals associated with quartz.^63^ Therefore, they indicate the presence of crystalline SiO_2_, originating from the filler—sand. The large group of peaks below the 500 cm^–1^ region likely originates from different T–O–T and ring-type structures, that may be associated with T–O–T bending vibrations and ring-related modes within aluminosilicate networks. Other peaks originate mostly from less cross-linked structures (largely unreacted raw materials), for example, bands near 873 and 911 cm^–1^, which are commonly attributed to hydroxylated species and residual aluminosilicate environments.^61^

The FTIR spectrum of a control sample with high degree of polymerization mold-cast sample shows strong similarities with test samples. The main difference is a shift of the T–O–T asymmetric stretching band toward lower wavenumbers by approximately 50 cm^–1^ relative to the printed tensile specimens. Such a shift is consistent with further reorganization of the aluminosilicate network and has been reported in geopolymer systems, where it is attributed to increased substitution of SiO_4_ units by AlO_4_ tetrahedra during gel formation.^64^ Divergent absorption features in the below 500 cm^–1^ region further indicate differences in the local network geometry.

## 4 Conclusions

This work demonstrates an approach aimed at lowering the high barrier to entry of geopolymer concrete additive manufacturing, particularly for early-stage research, by prioritizing system simplicity and high reproducibility. To this end, a low-cost and low-complexity modified tabletop extrusion system was shown to be suitable for geopolymer fine aggregate concrete additive manufacturing.

A commercial tabletop clay 3D printer was modified to produce fine aggregate, up to 1 mm particle size, geopolymer concrete. The mix was designed to reflect widely accepted geopolymerization chemical paths and tailored to the specific printer system by reduction of water-to-solid ratio for shape retention and the addition of Na-CMC as a viscosity modifier to ensure sufficient workability. The software and the general printing settings, resulting in consistent and successful geometries, were mapped and described in detail.

Directly measured 7-day tensile strength of 1.1 ± 0.2 MPa was achieved for dog-bone geometry samples printed with the designed manufacturing setup. This result is consistent with similar tensile measurements, indicating that the setup and mix design are capable of producing material with strength that is consistent with effective geopolymerization, when considered alongside spectroscopic evidence.

Multiscale defect characterization was utilized as an analytical tool to estimate both composite rheological and mix design microstructural parameters, revealing and discussing potential causes for the defects observed. ATR-FTIR spectroscopy further revealed absorption features characteristic of aluminosilicate networks commonly associated with the geopolymer gel formation.

Overall, the focus on reproducibility in this study provides a convenient and freely accessible first step for geopolymer 3D printing research. The small material volume required by the tabletop system is particularly advantageous for early-stage formulation screening, where raw material demand, waste generation, and iteration speed are critical practical constraints. By lowering practical barriers to experimentation, the presented approach supports wider exploration of geopolymer additive manufacturing and contributes to more sustainable construction practices.

## Supplementary Material



## Notes

Repository access information; bill of materials; printer assembly and calibration details; software overview and print parameter information; deposited line width correlation; drawings of custom-designed parts; printed geometries; dog-bone tensile testing procedure; mix preparation; and raw material chemical composition (PDF)

## References

[1] Davidovits, J. Synthesis of new high-temperature geo-polymers for reinforced plastics/composites; Society of Plastics Engineers: 1979, 151–154.

[2] Lehne, J. ; Preston, F. Making concrete change innovation in low-carbon cement and concrete; Chatham House, the Royal Institute of International Affairs: London, UK, 2018.

[3] Miller S. A., Horvath A., Monteiro P. J. M. (2016). Readily implementable
techniques
can cut annual CO_2_ emissions from the production of concrete
by over 20%. Environ. Res. Lett..

[4] van
Oss H. G., Padovani A. C. (2002). Cement manufacture and the environment:
Part I: Chemistry and technology. J. Ind. Ecol..

[5] van
Oss H. G., Padovani A. C. (2003). Cement manufacture and the environment:
Part II: Environmental challenges and opportunities. J. Ind. Ecol..

[6] Sbahieh S., McKay G., Al-Ghamdi S. G. (2023). Comprehensive
analysis of geopolymer
materials: Properties, environmental impacts, and applications. Materials.

[7] McLellan B. C., Williams R. P., Lay J., van Riessen A., Corder G. D. (2011). Costs and carbon emissions for geopolymer
pastes in
comparison to ordinary portland cement. J. Clean.
Prod..

[8] Albidah A., Alghannam M., Abbas H., Almusallam T., Al-Salloum Y. (2021). Characteristics of metakaolin-based geopolymer concrete
for different mix design parameters. J. Mater.
Res. Technol..

[9] Li J., Mailhiot S., Kantola A. M., Niu H., Sreenivasan H., Telkki V.-V., Kinnunen P. (2022). Longitudinal single-sided NMR study:
Silica-to-alumina ratio changes the reaction mechanism of geopolymer,
Cem. Concr. Res..

[10] Barbosa V. F. F., MacKenzie K. J. D., Thaumaturgo C. (2000). Synthesis
and characterisation of materials based on inorganic polymers of alumina
and silica: Sodium polysialate polymers. Int.
J. Inorg. Mater..

[11] Abdelrahman O., Garg N. (2022). Impact of Na/Al ratio on the extent
of alkali-activation reaction:
Non-linearity and diminishing returns. Front.
Chem..

[12] Pouhet R., Cyr M., Bucher R. (2019). Influence
of the initial water content in flash calcined
metakaolin-based geopolymer, Constr. Build.
Mater..

[13] Benavent V., Lahalle H., Patapy C., Renaudin G., Cyr M. (2022). Microstructural
evolution of a sodium metakaolin-based geopolymer paste in neutral
and CEM V basic environment. Cem. Concr. Res..

[14] Tuyan M., Zhang L. V., Nehdi M. L. (2020). Development
of sustainable preplaced
aggregate concrete with alkali-activated slag grout, Constr. Build. Mater..

[15] She Y., Wei K., Chen Y., Yu Q. (2024). Rheological properties and shootability
of sprayable geopolymer mortar, Constr. Build.
Mater..

[16] Adaloudis M., Bonnin Roca J. (2021). Sustainability
tradeoffs in the adoption of 3D concrete
printing in the construction industry. J. Clean.
Prod..

[17] Wijethunge, A. ; Samarasinghe, D. ; Le, A. ; Gajanayake, A. ; Atapattu, C. A systematic review on sustainable 3D concrete printing: Opportunities and challenges; Purdue University: 2025, 10.7771/3067-4883.1881.

[18] ISO/ASTM 52939:2023 Additive Manufacturing For Construction - Qualification Principles - Structural And Infrastructure Elements; International Organization for Standardization/ASTM International: Geneva, Switzerland/West Conshohocken, PA, USA, 2023.

[19] Martínez A., Miller S. A. (2023). A review of drivers for implementing
geopolymers in
construction: Codes and constructability. Resour.,Conserv.
Recycl..

[20] Khasawneh M. A. (2025). Geopolymer
concrete in construction projects: a review, Discov. Civ. Eng..

[21] Al-Raqeb H., Ghaffar S. H. (2025). Ghaffar, 3D concrete
printing in Kuwait: Stakeholder
insights for sustainable waste management solutions. Sustainability.

[22] Mandsberg N. K., Højgaard J., Joshi S. S., Nielsen L. H., Boisen A., Hwu E. T. (2021). Consumer-Grade
Inkjet Printer for Versatile and Precise
Chemical Deposition. ACS Omega.

[23] Tzu-Hsien, L. ; BoSheng, L. Overview of injection liquid printing with dredged-based material for concrete formwork. In IASS 2024 Zurich Symposium: Advanced Manufacturing and Materials; International Association for Shell and Spatial Structures (IASS), 2024.

[24] Barve P., Bahrami A., Shah S. (2023). Geopolymer
3D printing: A comprehensive
review on rheological and structural performance assessment, printing
process parameters, and microstructure. Front.
Mater..

[25] Chen K., Liu Q., Chen B., Zhang S., Ferrara L., Li W. (2024). Effect of
raw materials on the performance of 3D printing geopolymer: A review. J. Build. Eng..

[26] Sovetova M., Calautit J. K. (2024). Design, calibration and performance evaluation of a
small-scale 3D printer for accelerating research in additive manufacturing
in construction, Clean. Eng. Technol..

[27] Wittbrodt B. T., Glover A. G., Laureto J., Anzalone G. C., Oppliger D., Irwin J. L., Pearce J. M. (2013). Life-cycle
economic analysis of distributed
manufacturing with open-source 3-D printers. Mechatronics.

[28] Gomes
Gama J. F., Dias E. A., Aguiar Coelho R. M. G., Chagas A. M., Aguiar Coelho Nt J., Alves L. A. (2023). Development and
implementation of a significantly low-cost 3D bioprinter using recycled
scrap material. Front. Bioeng. Biotechnol..

[29] Thomas, D. S. ; Gilbert, S. W. Costs And Cost Effectiveness Of Additive Manufacturing; National Institute of Standards and Technology: Gaithersburg, MD, USA, 2014. 10.6028/NIST.SP.1176.

[30] Albar A., Chougan M., Al-Kheetan M. J., Swash M. R., Ghaffar S. H. (2020). Effective
extrusion-based 3D printing system design for cementitious-based materials. Results Eng..

[31] Ur
Rehman A., Sglavo V. M. (2020). 3D printing of geopolymer-based concrete
for building applications. Rapid Prototyping
J..

[32] Luhar S., Luhar I. (2020). Additive manufacturing
in the geopolymer construction technology:
A review, Open Constr. Build. Technol. J..

[33] Zhong H., Zhang M. (2022). 3D printing geopolymers:
A review. Cem. Concr.
Compos..

[34] Ricciotti L., Apicella A., Perrotta V., Aversa R. (2023). Geopolymer materials
for extrusion-based 3D-printing: A review. Polymers.

[35] Archez J., Texier-Mandoki N., Bourbon X., Caron J. F., Rossignol S. (2020). Adaptation
of the geopolymer composite formulation binder to the shaping process,
Mater. Today Commun..

[36] Tramontin
Souza M., Simão L., Guzi de Moraes E., Senff L., de Castro Pessôa J. R., Ribeiro M. J., Novaes de Oliveira A. P. (2021). Role of temperature in 3D printed
geopolymers: Evaluating rheology and buildability Mater. Mater. Lett..

[37] N’Cho W. C., Gharzouni A., Sobrados I., Jouin J., Aimable A., Rossignol S. (2025). Extrudability of geopolymers and control of the formed
networks by zeta potential and NMR spectroscopy. Int. J. Ceram. Eng. Sci..

[38] Agnoli E., Ciapponi R., Levi M., Turri S. (2019). Additive manufacturing
of geopolymers modified with microalgal biomass biofiller from wastewater
treatment plants. Materials.

[39] Alghamdi H., Neithalath N. (2019). Synthesis and characterization of 3D-printable geopolymeric
foams for thermally efficient building envelope materials. Cem. Concr. Compos..

[40] Tan Z., Bernal S. A., Provis J. L. (2017). Reproducible mini-slump test procedure
for measuring the yield stress of cementitious pastes. Mater. Struct..

[41] Yang J., Su J., Chen B., Luo X., Shen X. (2018). The optimized design
of dog-bones for tensile test of ultra-high-performance concrete. Eng. Mech..

[42] ISO 14688–1:2018 Geotechnical Investigation And Testing - Identification And Classification Of Soil - Part 1: identification And Description; International Organization for Standardization Geneva, Switzerland, 2018.

[43] Chen C., Zhang X., Hao H. (2023). Dynamic tensile properties of geopolymer
concrete and fibre reinforced geopolymer concrete. Constr. Build. Mater..

[44] Guades E. J. (2016). Experimental
investigation of the compressive and tensile strengths of geopolymer
mortar: The effect of sand/fly ash (S/FA) ratio. Constr. Build. Mater.

[45] Al-Majidi M. H., Lampropoulos A., Cundy A. B. (2017). Tensile properties of a novel fibre
reinforced geopolymer composite with enhanced strain hardening characteristics. Compos. Struct..

[46] Huang D., Chen X., Liu Z., Lu Y., Li S. (2025). Microwire
steel fiber-reinforced geopolymer recycled aggregate concrete under
uniaxial compression and tension: Mechanical properties and constitutive
models. Constr. Build. Mater..

[47] Kozub B., Sitarz M., Gądek S., Ziejewska C., Mróz K., Hager I. (2024). Upscaling of copper
slag-based geopolymer
to 3D printing technology. Materials.

[48] Mermerdaş K., Khalid L. W., Bzeni D. K., İpek S., Jawad D. J. (2024). Tensile strength and durability performances-based
evaluation of 3D-printed and mold-cast fibrous geopolymer composites
produced at various alkaline activator combinations. J. Build. Eng..

[49] Ahmed G. H. (2023). A review
of “3D concrete printing”: Materials and process characterization,
economic considerations and environmental sustainability. J. Build. Eng..

[50] Agegn A. A., Regassa Y., Angassa K., Mekonnen K. N. (2026). Systematic review
on 3D concrete printing technology: Breakthroughs and challenges. Discovery Civ. Eng.

[51] Chen Y., Zhang L., Wei K., Gao H., Liu Z., She Y., Chen F., Gao H., Yu Q. (2024). Rheology control and
shrinkage mitigation of 3D printed geopolymer concrete using nanocellulose
and magnesium oxide. Constr. Build. Mater.

[52] Potter P. E. (1978). Petrology
and chemistry of modern big river sands. J.
Geo..

[53] Demie S., Nuruddin M. F., Shafiq N. (2013). Effects of
micro-structure characteristics
of interfacial transition zone on the compressive strength of self-compacting
geopolymer concrete. Constr. Build. Mater..

[54] Ren X., Zhang L. (2018). Experimental study
of interfacial transition zones between geopolymer
binder and recycled aggregate. Constr. Build.
Mater..

[55] Fang G., Wang Q., Zhang M. (2021). Micromechanical analysis of interfacial
transition zone in alkali-activated fly ash-slag concrete. Cem. Concr. Compos.

[56] Dan H., Li M., Tan J., Dan H., Ma Z., Ma S. (2023). Nano- and
microscale characterization for interfacial transition zone of geopolymer
stabilized recycled aggregate of asphalt pavement. Constr. Build. Mater..

[57] Ahmari S., Zhang L., Zhang J. (2012). Effects of activator type/concentration
and curing temperature on alkali-activated binder based on copper
mine tailings. J. Mater. Sci..

[58] He P., Wang M., Fu S., Jia D., Yan S., Yuan J., Xu J., Wang P., Zhou Y. (2016). Effects of
Si/Al ratio on the structure and properties of metakaolin based geopolymer. Ceram. Int..

[59] Davidovits, J. Geopolymer chemistry and applications; Geopolymer Institute, 2020.

[60] Rahier H., Wastiels J., Biesemans M., Willlem R., Van Assche G., Van Mele B. (2007). Reaction mechanism,
kinetics and high temperature transformations
of geopolymers. J. Mater. Sci..

[61] Li Z., Zhang S., Zuo Y., Chen W., Ye G. (2019). Chemical deformation
of metakaolin based geopolyme. Cem. Concr. Res..

[62] Gonçalves D. K.
C., Lana S. L. B., Sales R. B. C., Aguilar M. T. P. (2022). Study of metakaolins
with different amorphities and particle sizes activated by KOH and
K2SiO3. Case Stud. Constr. Mater..

[63] Farmer, V. C. The infrared spectra of minerals; Mineralogical Society, 1974. 10.1180/mono-4.

[64] Alehyen S., Achouri M. E., Taibi M. (2017). Characterization,
microstructure
and properties of fly ash-based geopolymer. J. Mater. Environ. Sci..

