# Supplementary material for: Cost-Effective 3D Printing of Geopolymer Concrete Enhanced by Na-CMC as a Viscosity Modifier
Source: ACS Omega. 2026 Jul 20;11(30):45092–103. doi: 10.1021/acsomega.6c03385 (PMC13448947; doi:10.1021/acsomega.6c03385)
Supplement: Supplementary file 1 [file ao6c03385_si_001.pdf]

Supporting Information

Cost-effective 3D Printing of Geopolymer Concrete Enhanced by Na-CMC as a Viscosity Modifier

Siim Koor[a*], Hans Priks[a], Tarmo Tamm[a]

[a] Institute of Technology, University of Tartu, 1 Nooruse St., 50411 Tartu, Estonia.

*siim.koor@ut.ee

Table of contents

SI section 1 – Access to repositories 2

SI section 2 – Bill of materials 3

SI section 3 – Printer assembly 4

Extruder head 4

Hose system and piping 5

Syringe tube frame 6

Z-axis calibration 6

Software overview 9

Deposited line width correlation 10

Engineering Drawings of custom-designed parts 11

SI section 4 – Printed geometries 14

SI section 5 – Dog-bone tensile testing 16

Sample printing and curing 16

Tensile testing 17

SI section 6 – Mix preparation 18

SI section 7 – Chemical composition of raw materials 21

SI section 1 – Access to repositories

The complete supplementary repository (v1.0.1) is archived at Zenodo:
https://doi.org/10.5281/zenodo.18623879

The live development repository is available at GitHub:

https://github.com/SiimKoor/low-cost-geopolymer-3dp-paper-2026

SI section 2 – Bill of materials

The total cost of printer and materials used for modifications was 571€ at the time of purchase in 2024, taxes included. Cost's detailed breakdown and bill of materials (BOM) can be found in Table S1. Notably, 91% of total costs came from the purchase of the printer itself and 9% from other materials. The BOM describes the minimal consumption of materials needed to construct the printer system, rather than the amount of materials that was purchased.

Table S1. Bill of materials.

Item	Material	Unit	Amount	Cost per unit (€)	Total cost (€)
3D printer					
Tronxy Moore 2 Pro Ceramic & Clay 3D printer		pc.	1	517.41	517.41
Hose system, nozzles and extruder head					
Pneumatic fitting Ø10mm-1/4" male	AISI 304	pc.	2	3.76	7.52
Ball valve 1/4" male to female	Ni plated brass	pc.	1	5.37	5.37
Hose barb fitting 7mm-G1/4" male	AISI 316	pc.	1	1.50	1.50
Hose barb fitting 9mm-G1/4" male	AISI 316	pc.	1	1.54	1.54
Hose barb fitting 10mm-G1/4" male	AISI 316	pc.	1	1.33	1.33
Hose barb fitting 12mm-G1/4" male	AISI 316	pc.	1	1.75	1.75
Threaded pipe, Ø15mm-1/4" female	E235+N	m	1	7.32	7.32
Hose, ID Ø8mm, OD Ø10mm	PVC	m	2	0.54	1.08
Custom 3D printed nozzle frame	Prusament PLA	kg	0.092	29.99	2.76
Extruder system frame					
Al T-slot profile 30x30mm	EN AW-6063-T5	m	2.8	4.5	12.6
Plywood plate	HPL-faced plywood	m^2	0.08	25.34	2.03
Custom 3D printed joints	Prusament PLA	kg	0.014	29.99	0.42
Bolts, nuts, fittings, common joints					
Materials	AISI 304				10.00 est.

SI section 3 – Printer assembly

The following describes printer assembly and performed modifications in sufficient detail to ensure complete reproducibility. Figure S1 illustrates general changes.

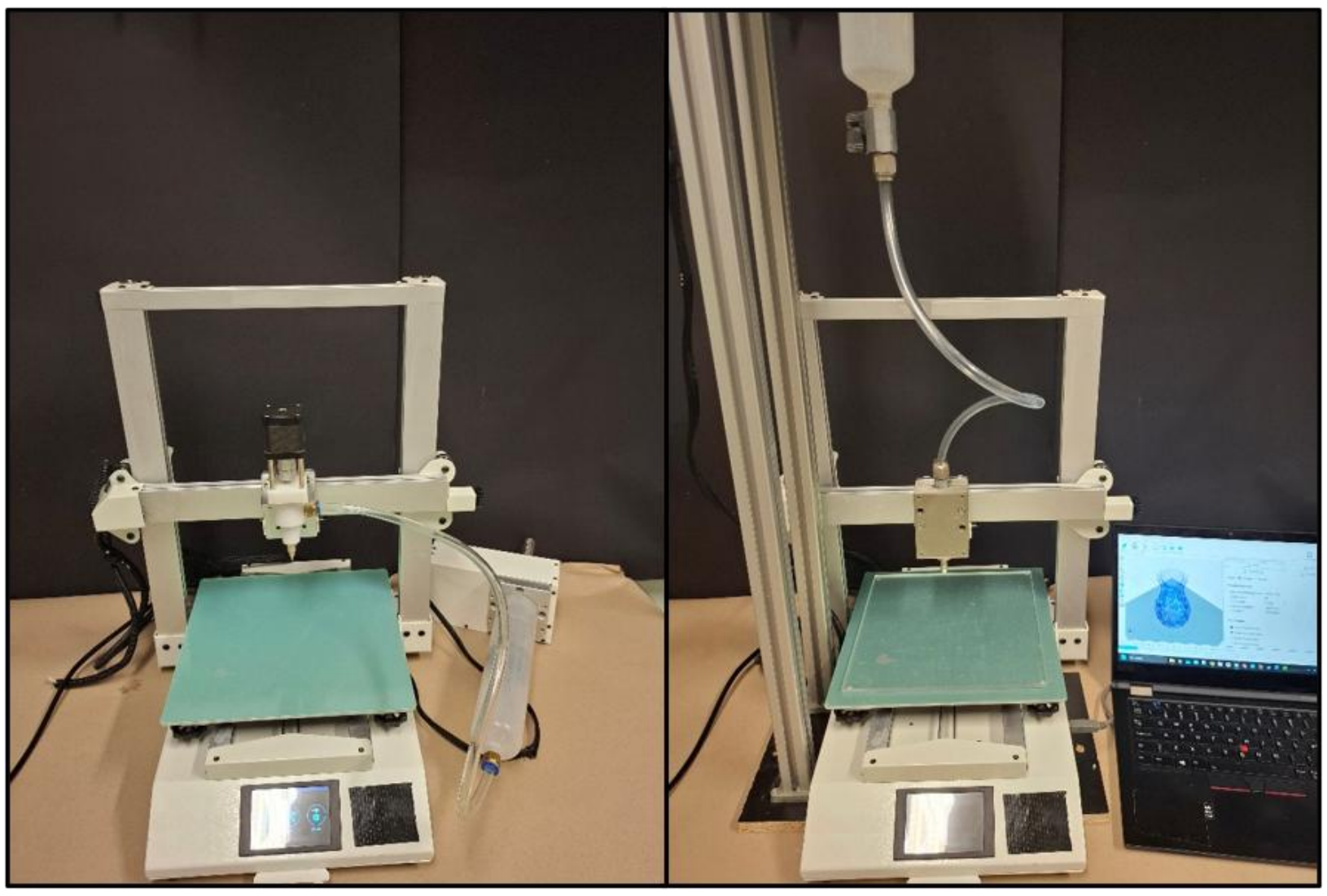

Figure S1. Printer in default configuration and in modified setup.

Extruder head

Figure S2 describes custom designed printer head assembly and Figure S3 compatible wedge system for nozzle angle adjustment. The central piping is entirely fixed by friction gripping, utilising 2-4x M4x40 mm bolts for tensioning. The two main printer head parts have a cylindrical pipe shaped cavity with slightly smaller diameter than the pipe itself, ensuring high friction and nozzle head 90° angle relative to the x-axis. The wedge system allows to align nozzle head in a 90° angle relative to the y-axis. Detailed drawings and models can be found below and/or in associated GitHub repository.

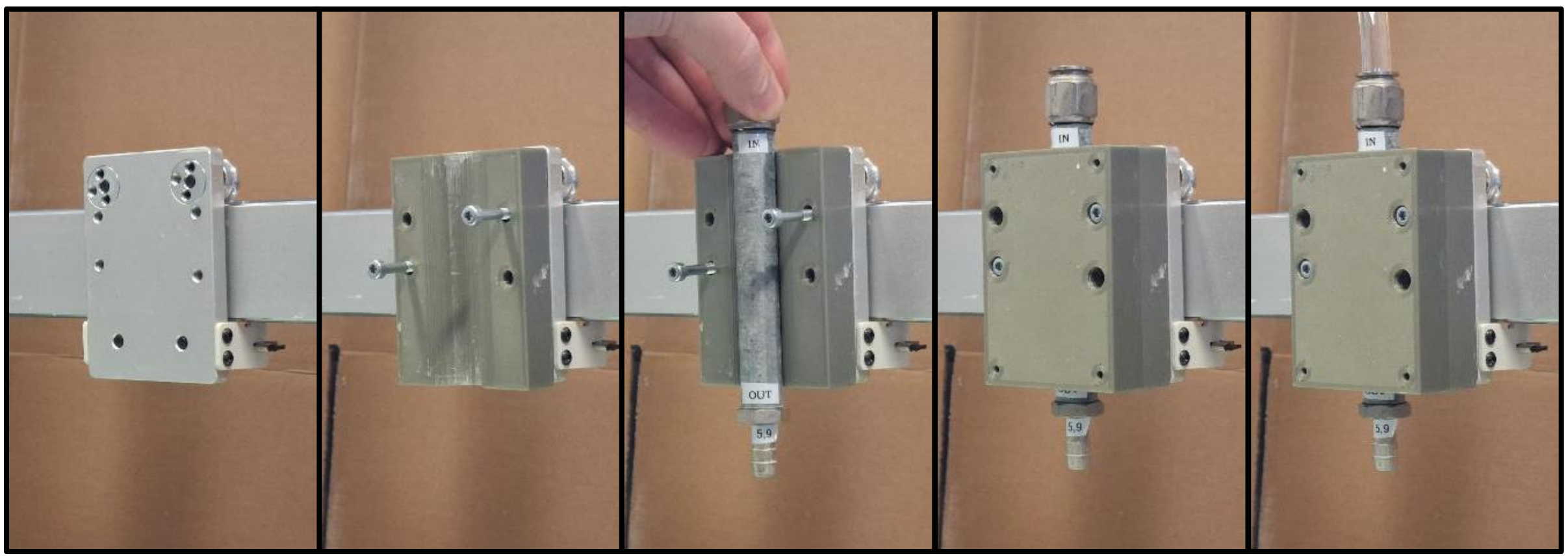

Figure S2. Custom print head assembly.

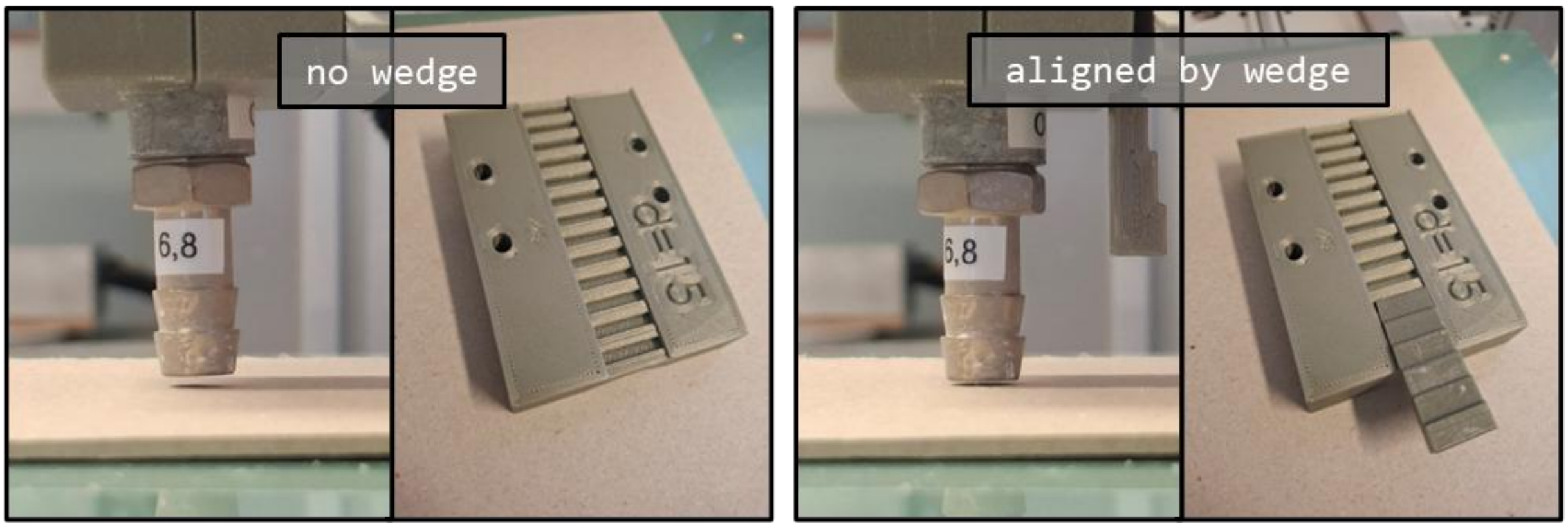

Figure S3. Nozzle-angle adjustment wedge.

Hose system and piping

Stainless steel piping was used for extruder head (Figure S4). Threads were machined into pipes using a lathe. Since thread cutting needs to be highly precise, the use of industrially machined threaded pipe is recommended, if possible. Threading allows for the convenient use of different diameter barb fittings as nozzle heads and the attachment of push-fit hose system (Figure S5). The central hole of hose push-fittings were redrilled into larger round shape to mitigate bottleneck effects at hose system junctions and reduce non-laminar flow effects.

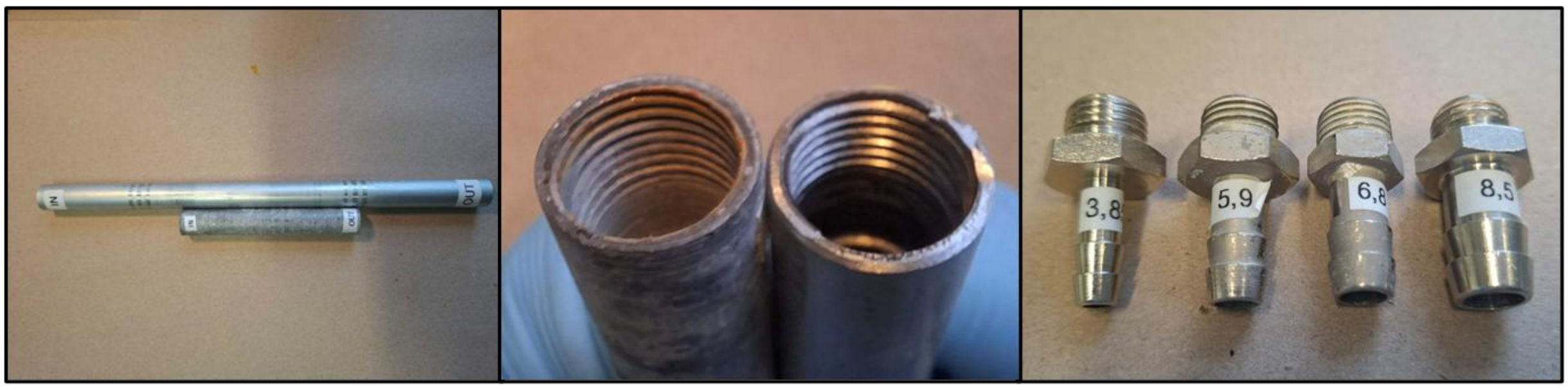

Figure S4. Extruder head piping and changeable different sized nozzles.

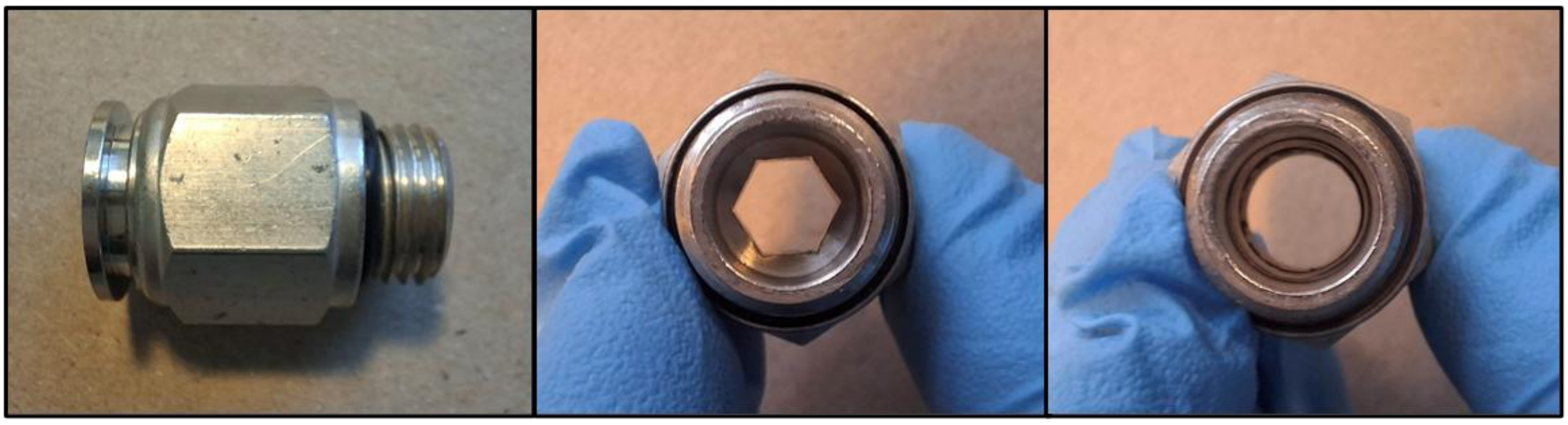

Figure S5. 10mm hose to 1/4" thread push-fitting.

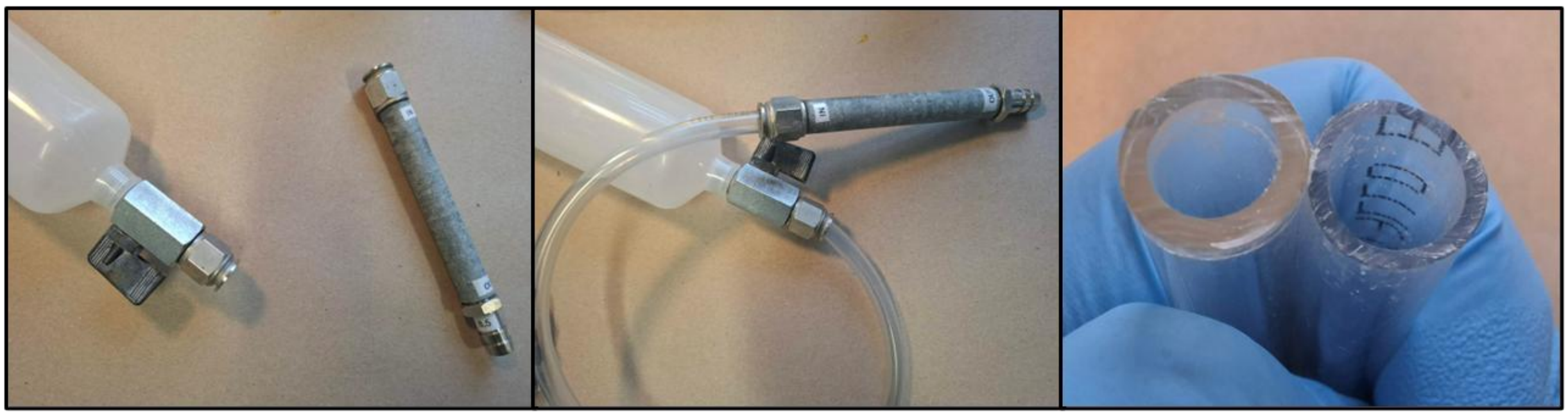

Figure S6. Hose system assembly. Inner diameter 6 mm (left) and 8 mm (right) hoses on the rightmost image.

Syringe tube frame

The material storage and plunger-extruder (syringe tube) frame was designed by utilising readily available materials (Figure S7). Main goal of the frame was to fix the system separately from the printer and above the printer itself in a downward facing orientation to ensure that the material exclusively moves downwards in the z-axis direction. For construction 30x30 mm aluminium T-slot profile, fixed with L-type corner brackets and M8x15mm bolts and nuts, was used.

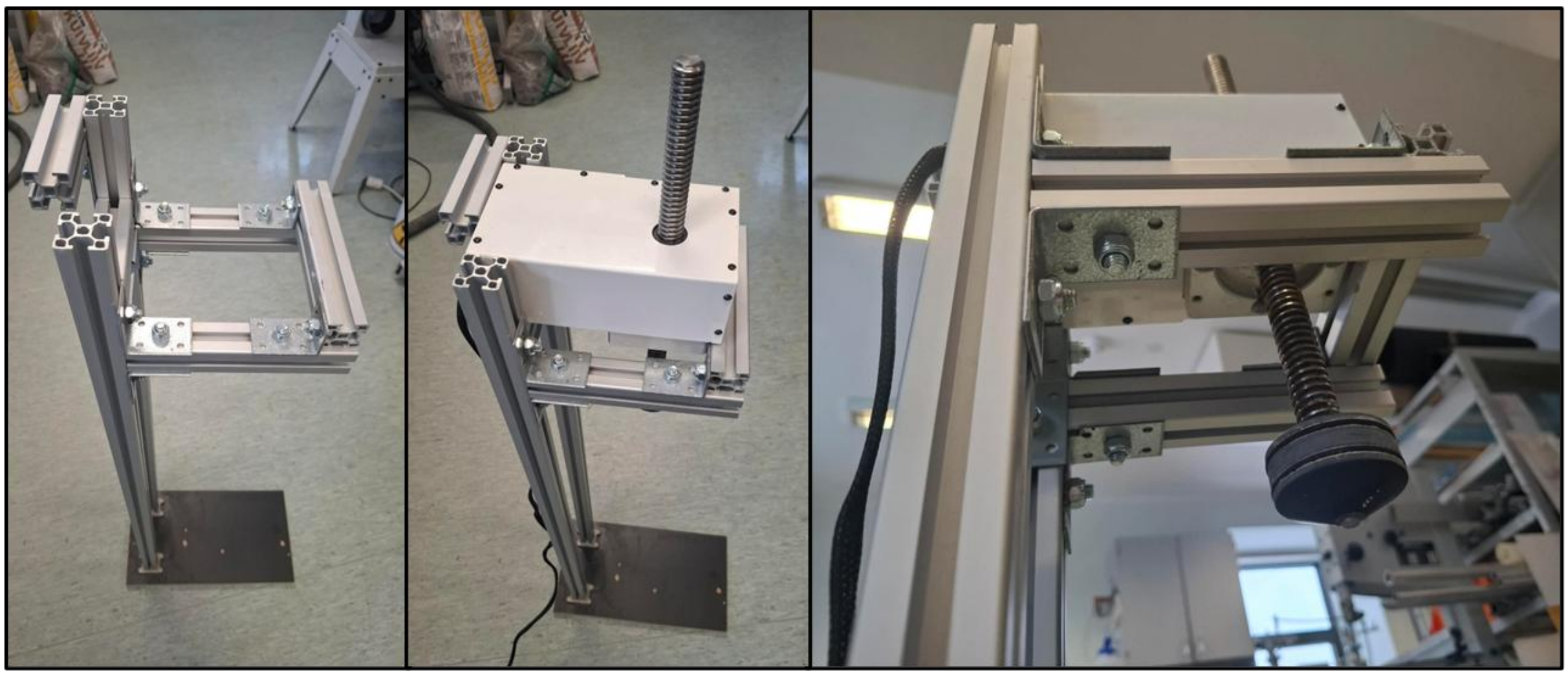

Figure S7. Syringe tube and plunger-extruder frame.

Z-axis calibration

Initial calibration of hardware should be performed according to default printer manual.

For the custom printer head, simple calibration is needed after every disassembly to align z-axis coordinates with software preset values. Repetier-Host or other live control is recommended for this task. The calibration was performed as follows:

Print sheet was placed upon the print bed (Figure S8). 3 to 6 mm polycarbonate sheet offers lightweight, yet sufficiently strong option, that has sufficient adhesion to grip deposited material but later allows for cured material to be easily removed.

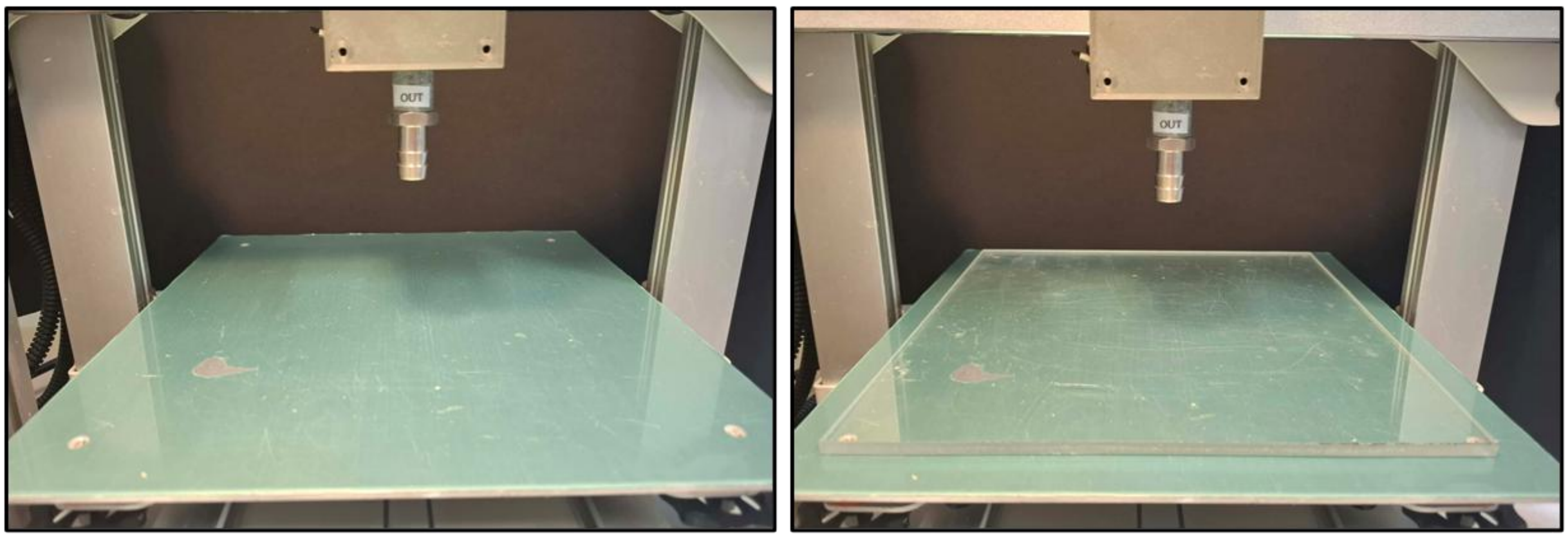

Figure S8. Print sheet placement.

Extruder head piping (Figure S9) with nozzle attached was fixed to the printer head frame, but the fixing bolts were not fully tightened. The pipe and nozzle should have sufficient freedom to move themselves if any force is applied.

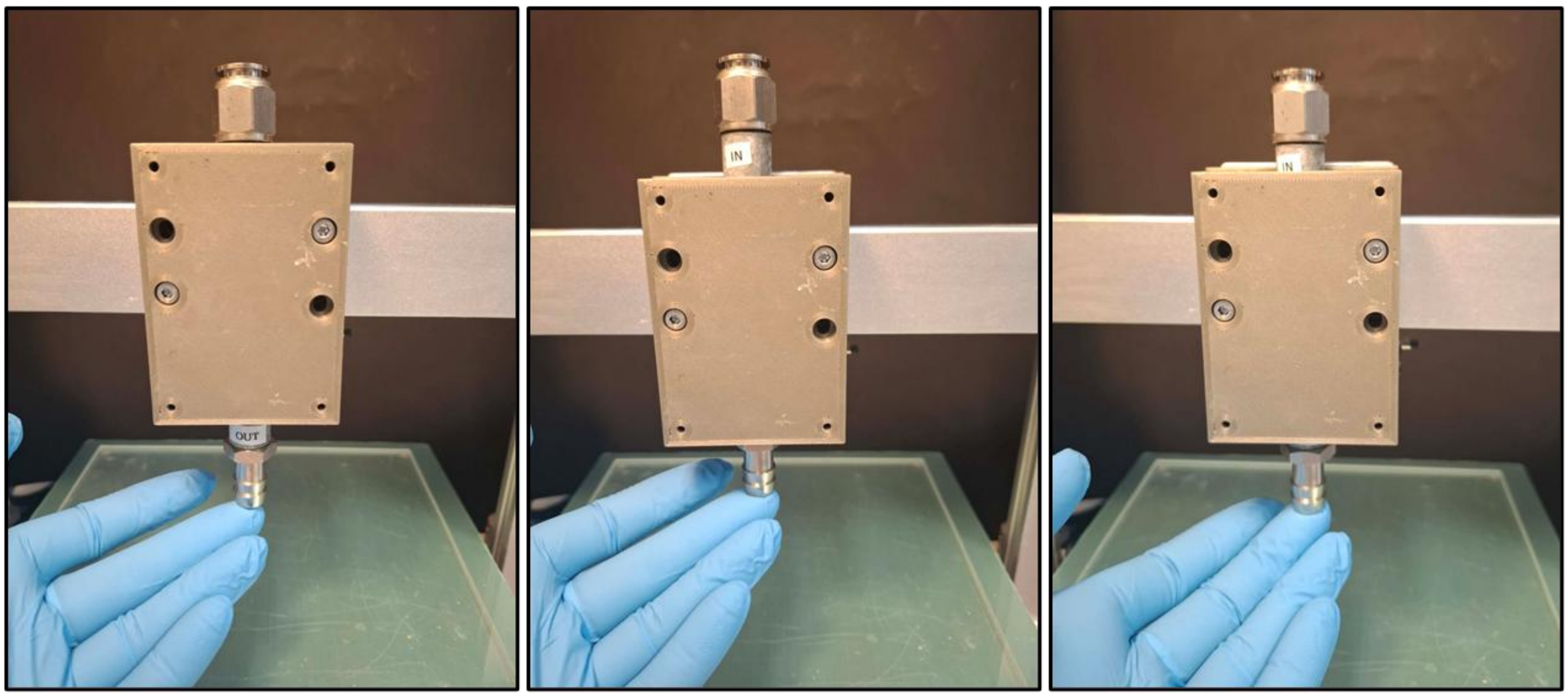

Figure S9. Extruder head piping attachment.

Z-axis was set to move into 0-position in the software. It should be made sure that the piping and nozzle have enough clearance to move and not be pushed into the print bed by the printer head frame (Figure S10).

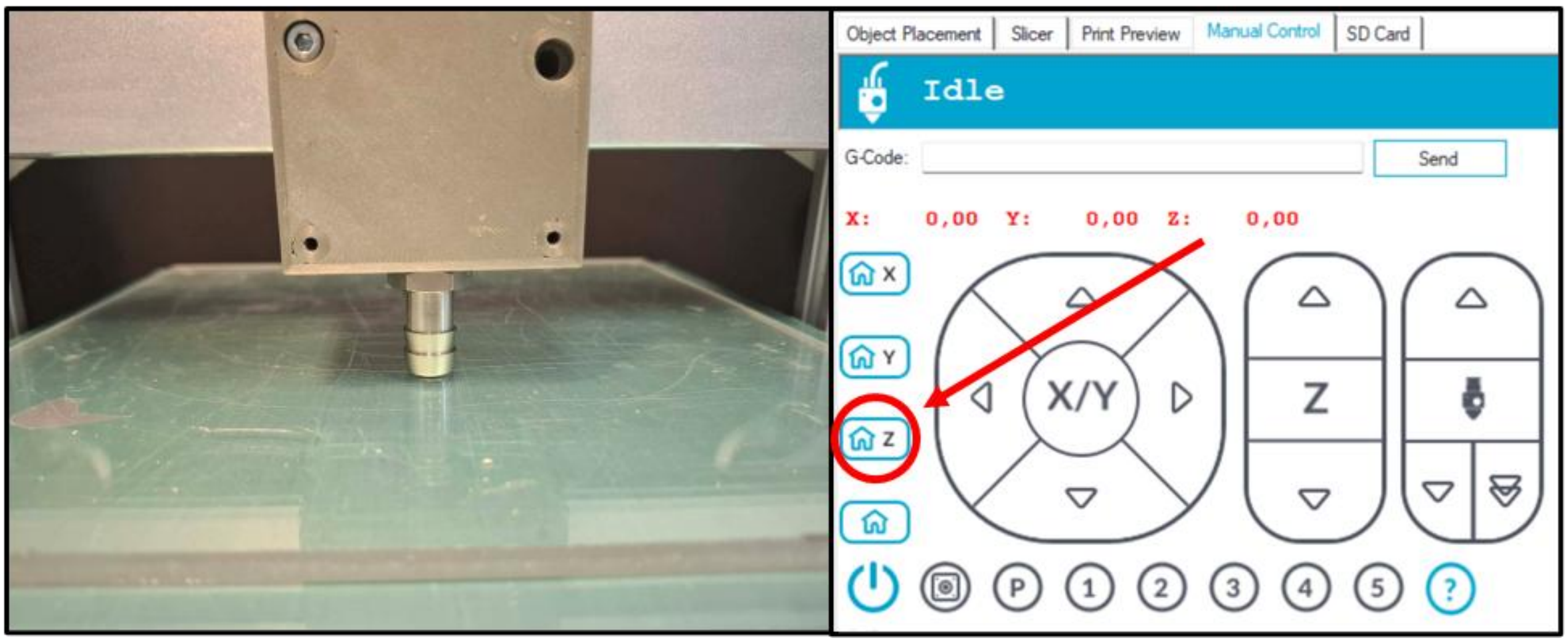

Figure S10. Z-axis 0-position configuration – part 1.

A 3-5 mm clearance between the print sheet and nozzle opening was fixed. Four layers of paper were used for this task for convenience. Nozzle can fully rest upon the sheets of paper. Then the printer head frame bolts were fully tightened, fixing the piping and nozzle (Figure S11). The nozzle opening was now aligned with 0-value set in the printer software. The paper was removed and z-axis considered as calibrated.

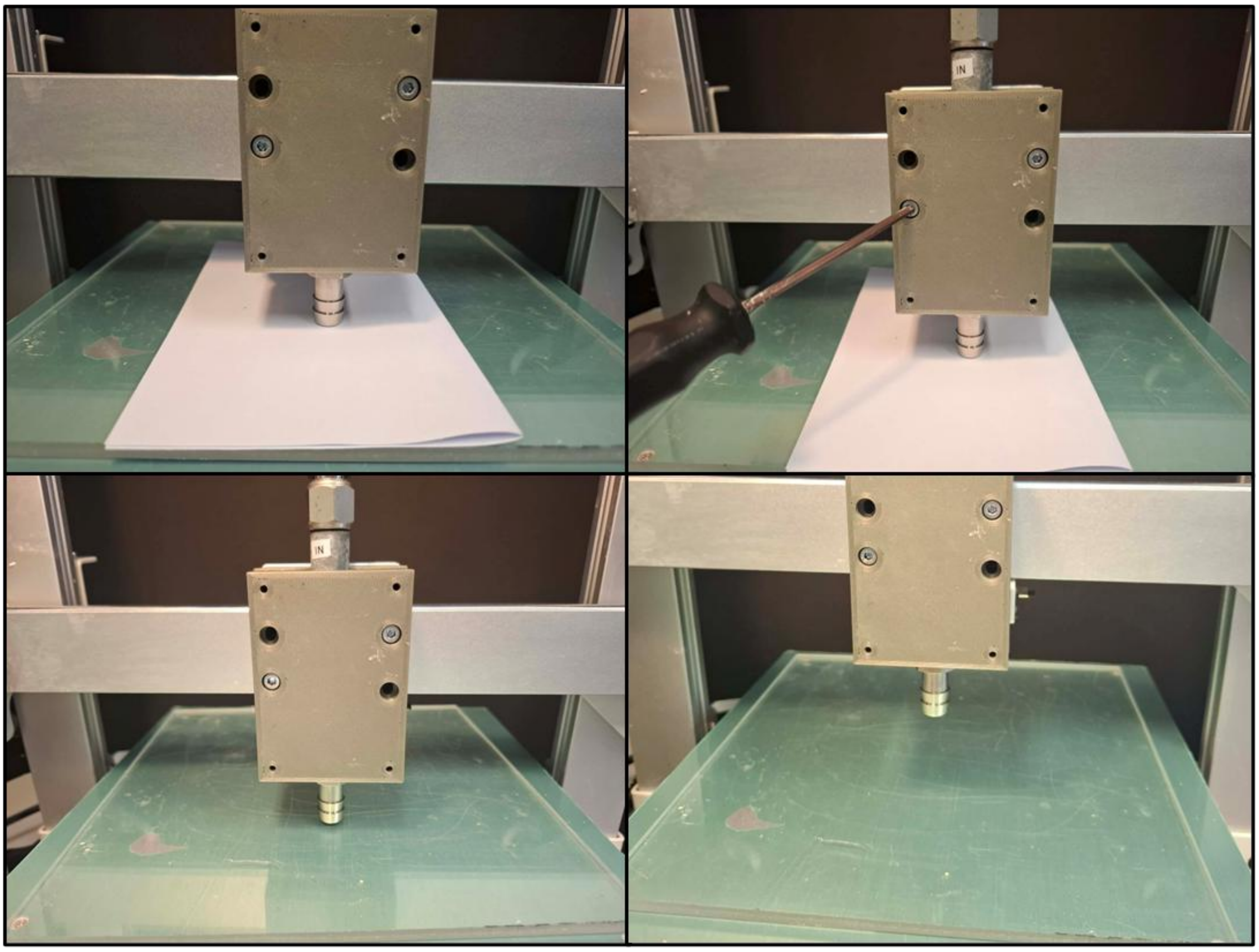

Figure S11. Z-axis 0-position configuration – part 2.

Software overview

During print code running machine extrusion parameter (home > tool > materials) should be set to "10.000 + medium".

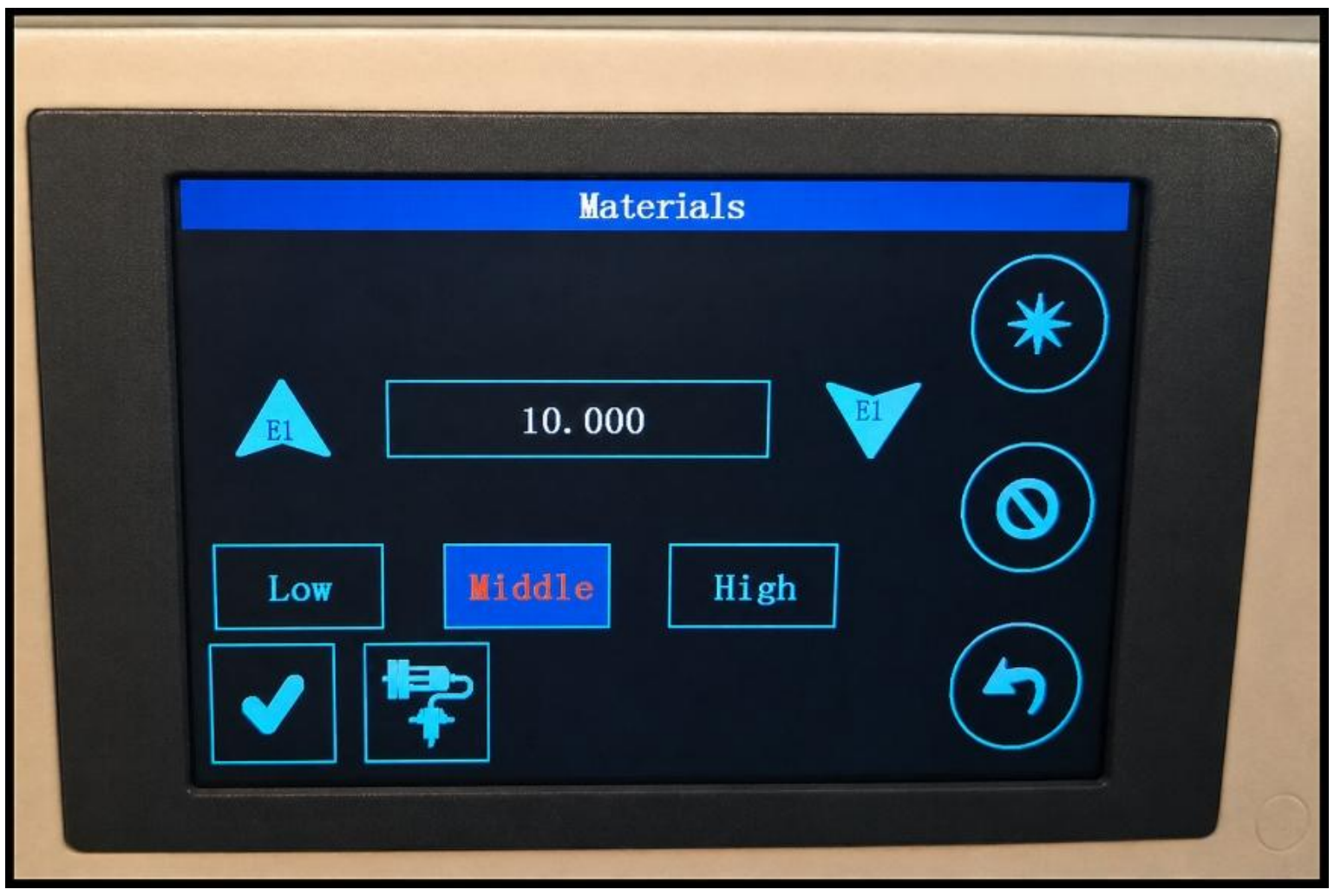

Figure S12. Machine software preset.

For printer operation PrusaSlicer v2.9.0 and Repetier-Host v2.3.2 were used. Data packages containing software presets can be imported from the supplementary repository.

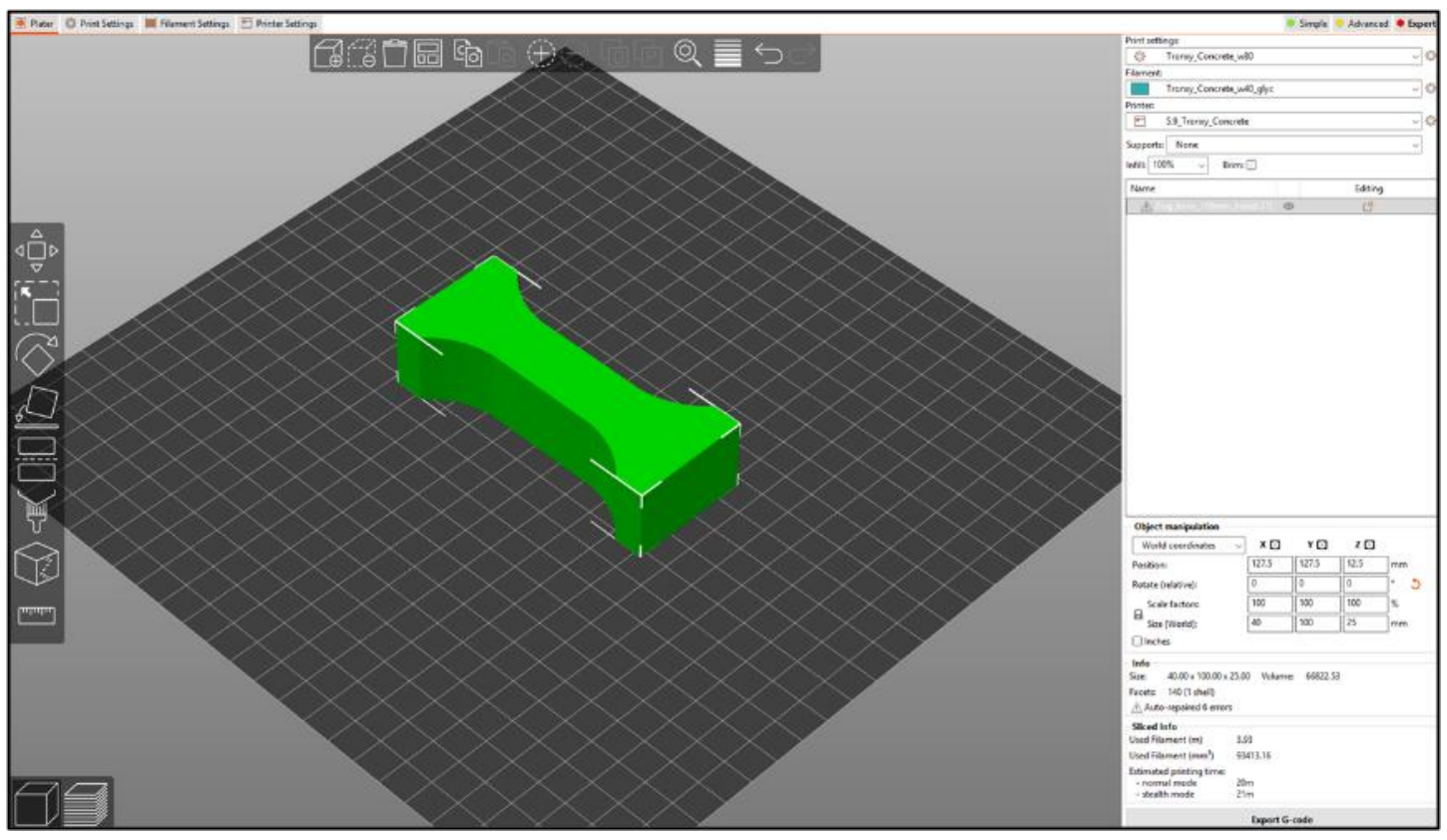

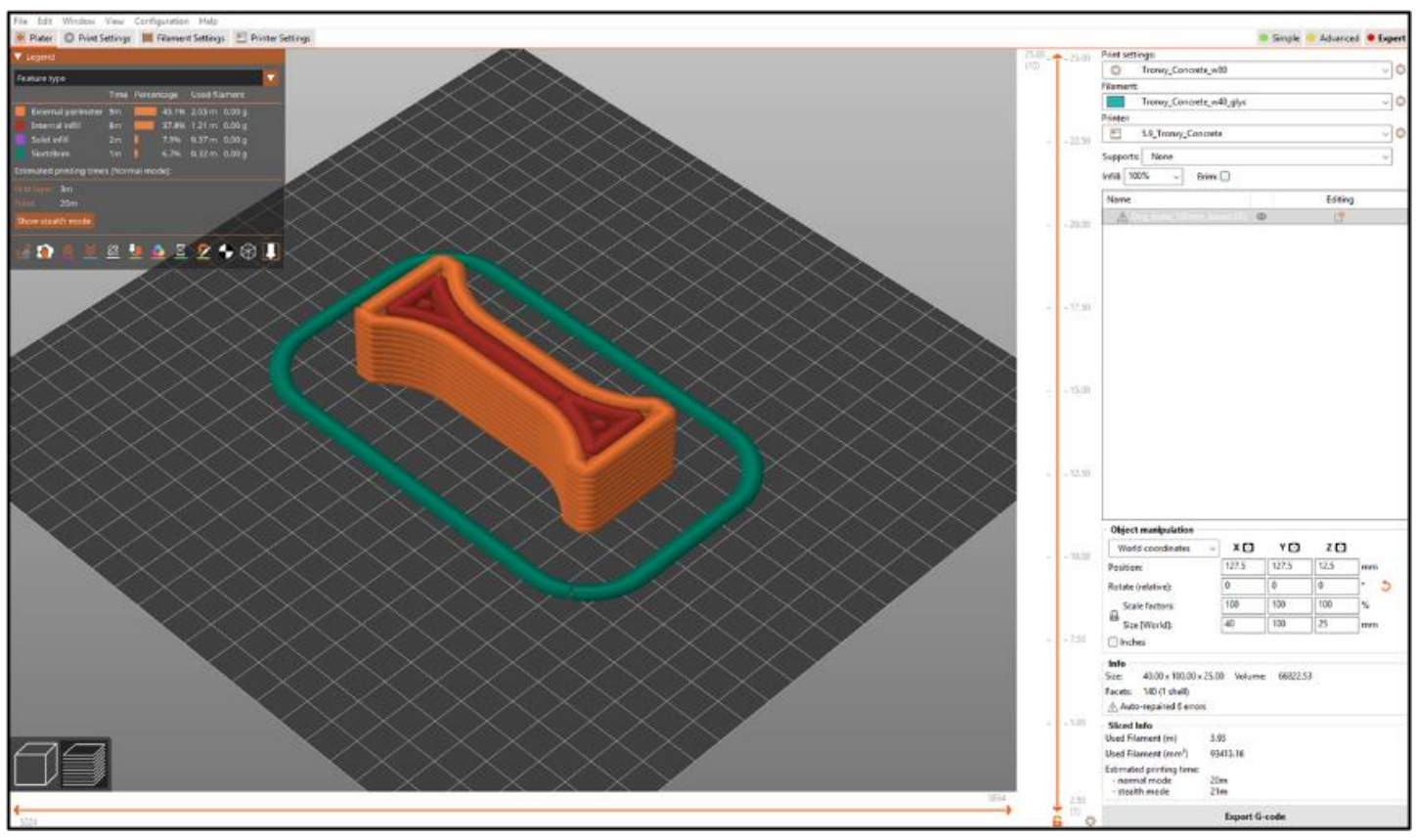

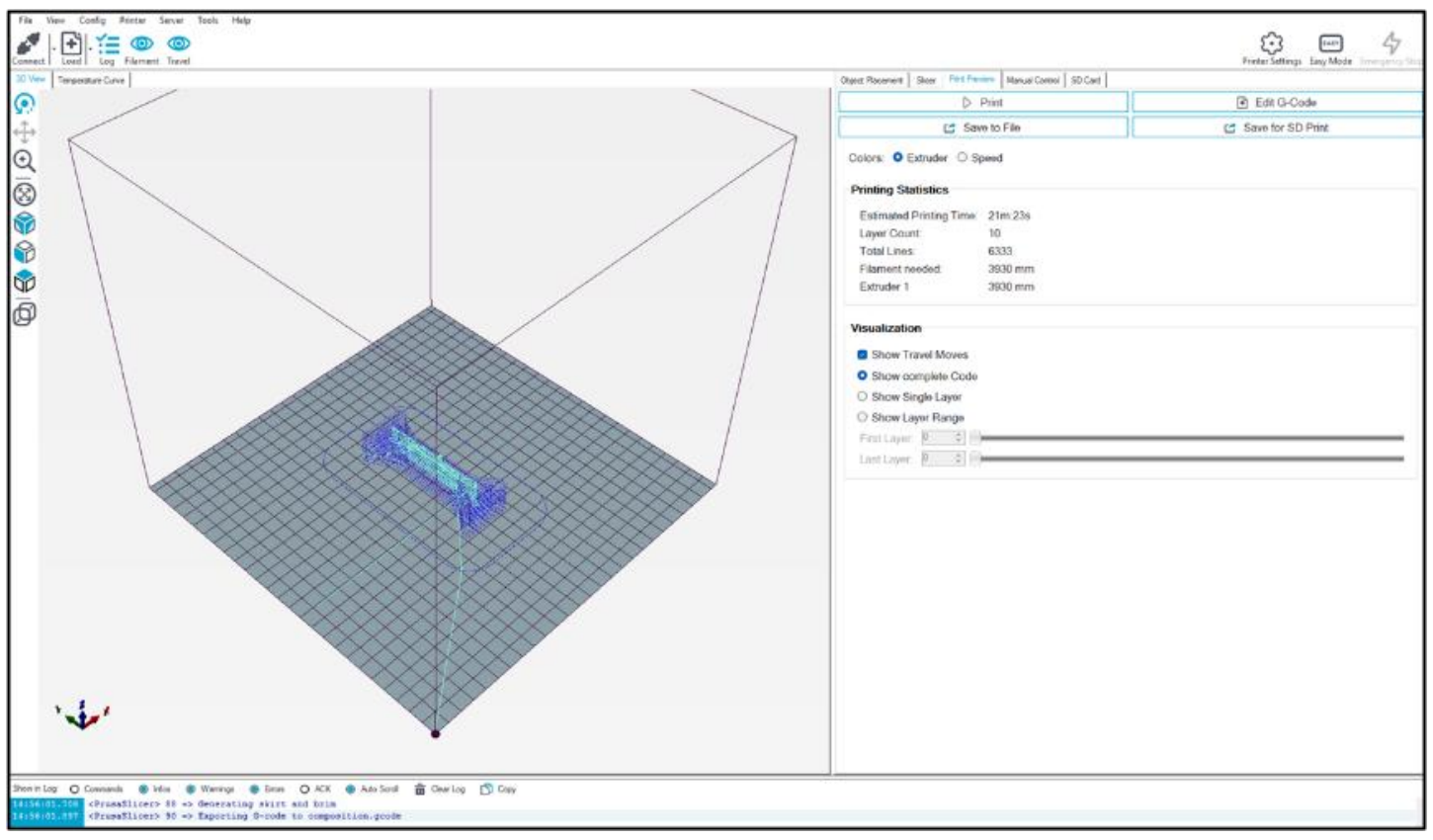

Figure S13. Excamples of dog-bone tensile testing specimens depicted in printing softwares at different stages.

Deposited line width correlation

Figure S14 describes deposited line width as a function of volumetric flow rate set in slicer software or live control. The linear fit parameters and coefficient of determination can be observed as such:

$$y = 3.0222x + 2.7844$$

$$R^2 = 0.9858$$

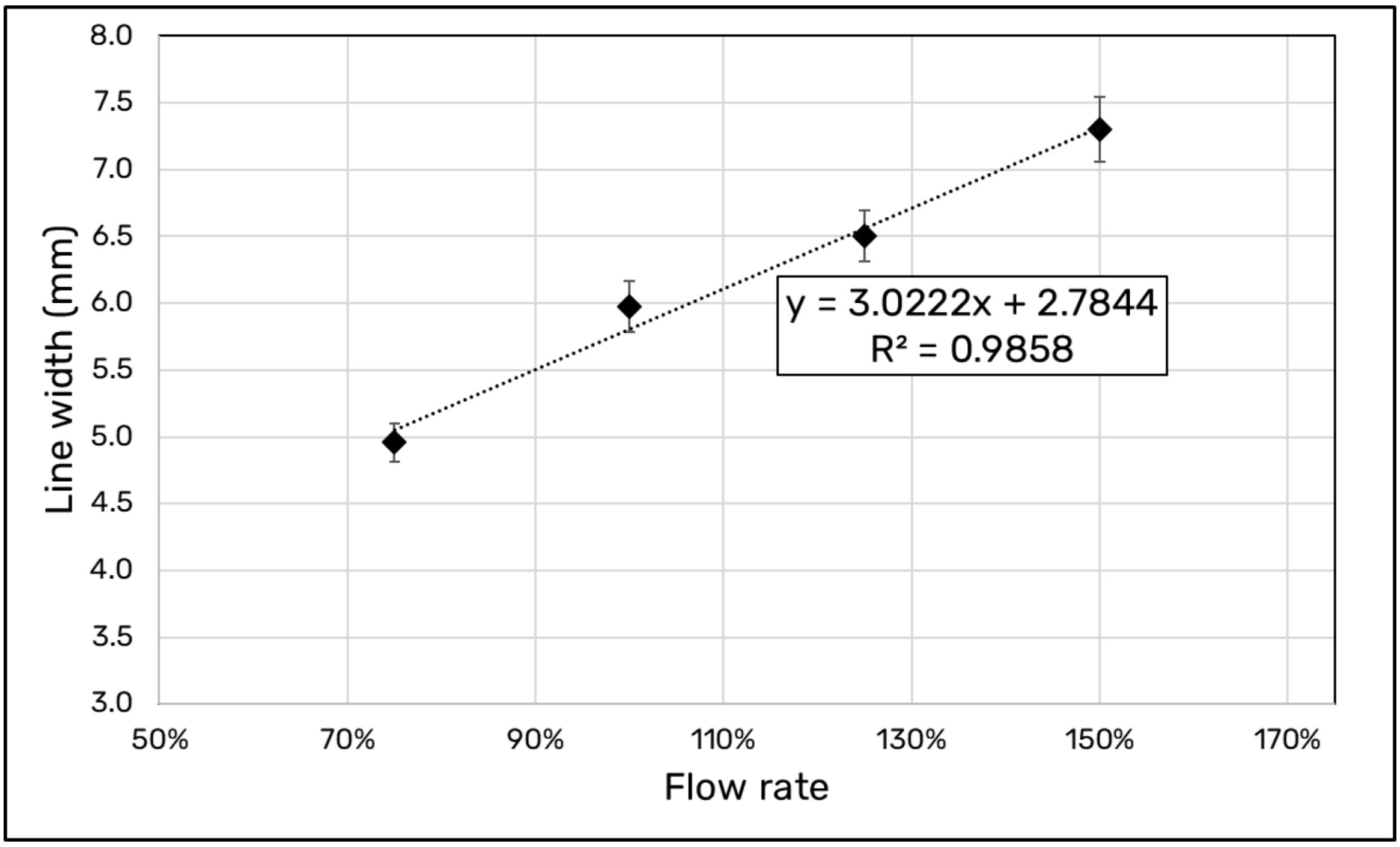

Figure S14. Deposited line width as a function of volumetric flow rate using 5.9 mm nozzle.

Engineering Drawings of custom-designed parts

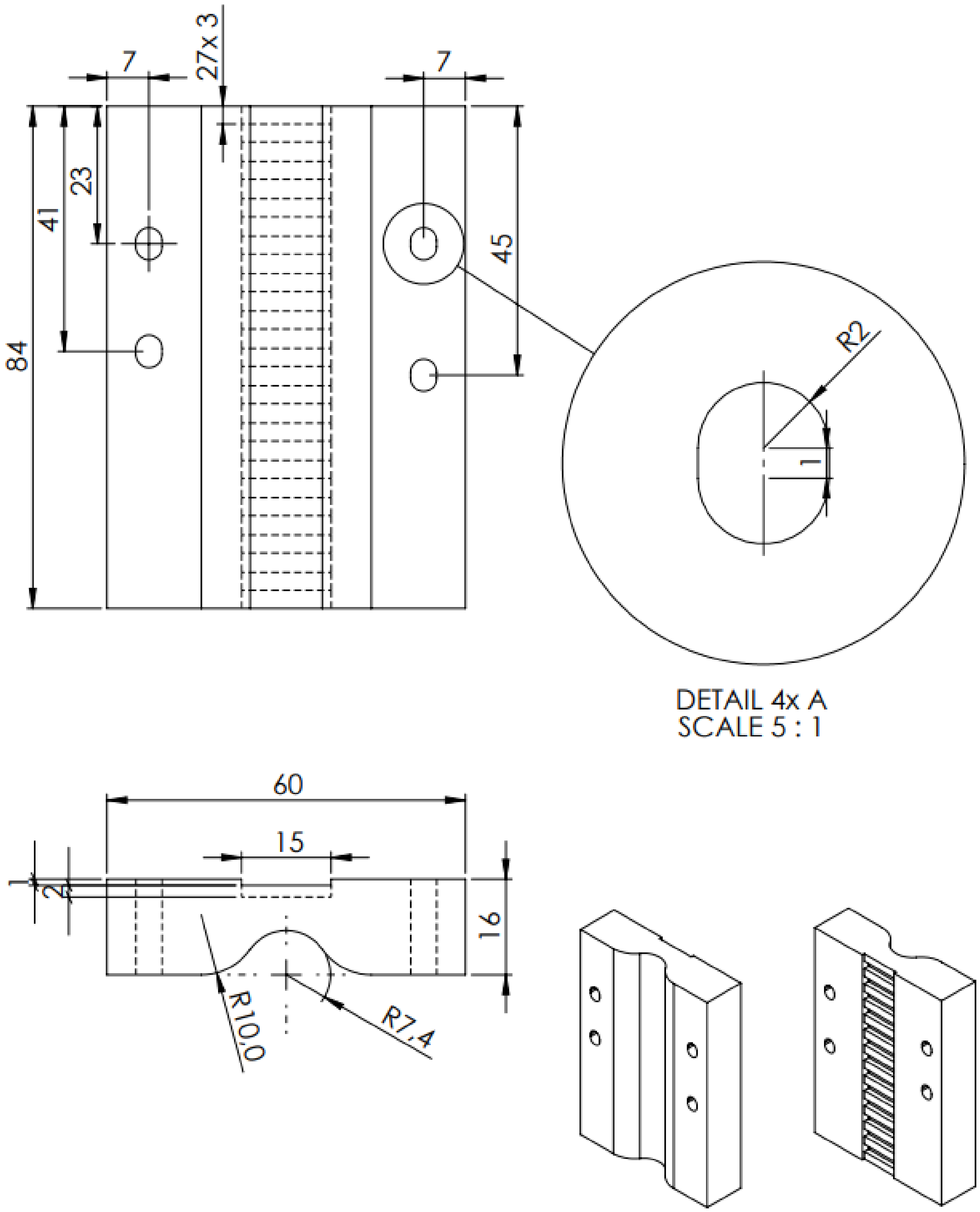

Engineering Drawing S1. Extruder head attachment part A. Material PLA. Models available at associated GitHub repository.

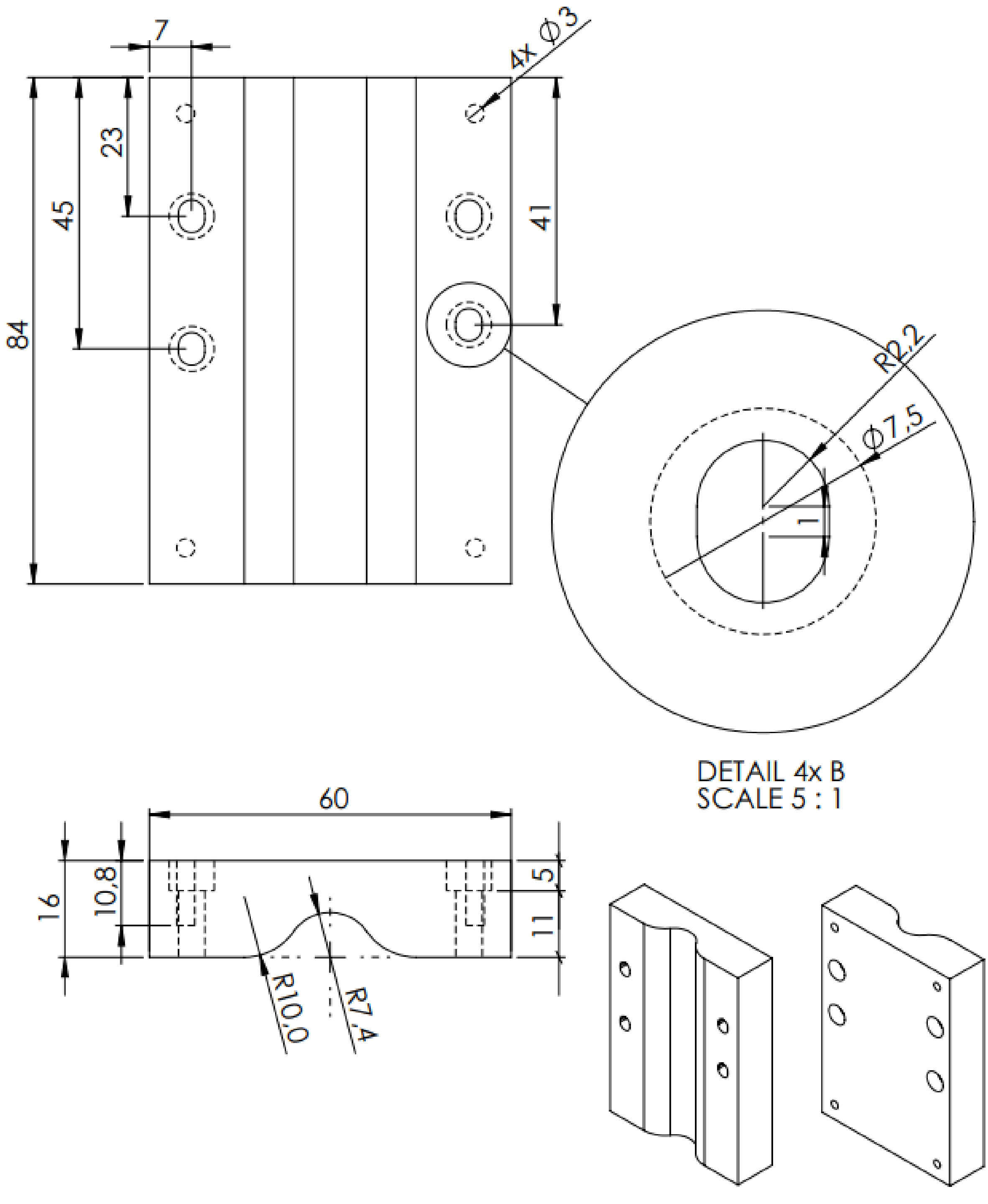

Engineering Drawing S2. Extruder head attachment part B. Material PLA. Models available at associated GitHub repository.

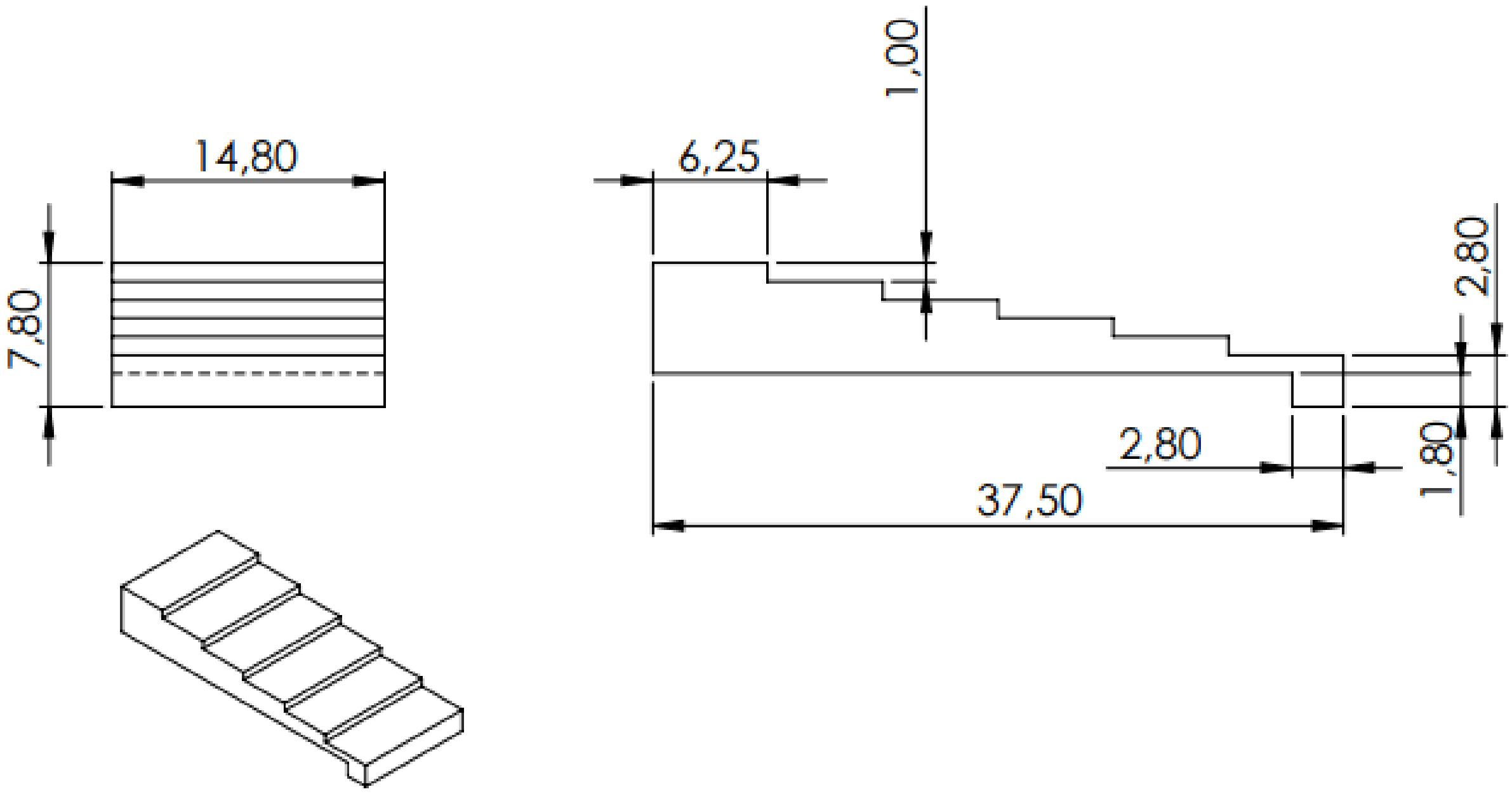

Engineering Drawing S3. Extruder head attachment wedge part. Material PLA. Models available at associated GitHub repository.

SI section 4 – Printed geometries

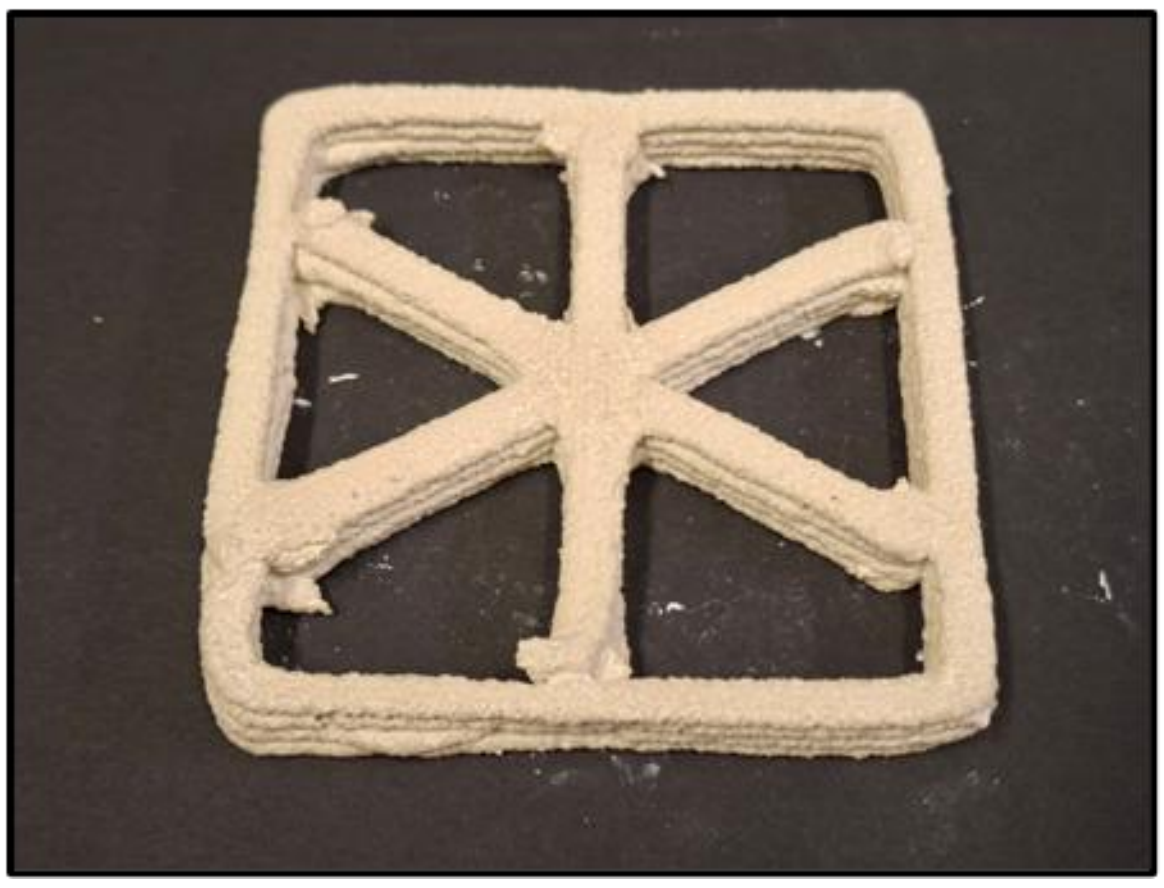
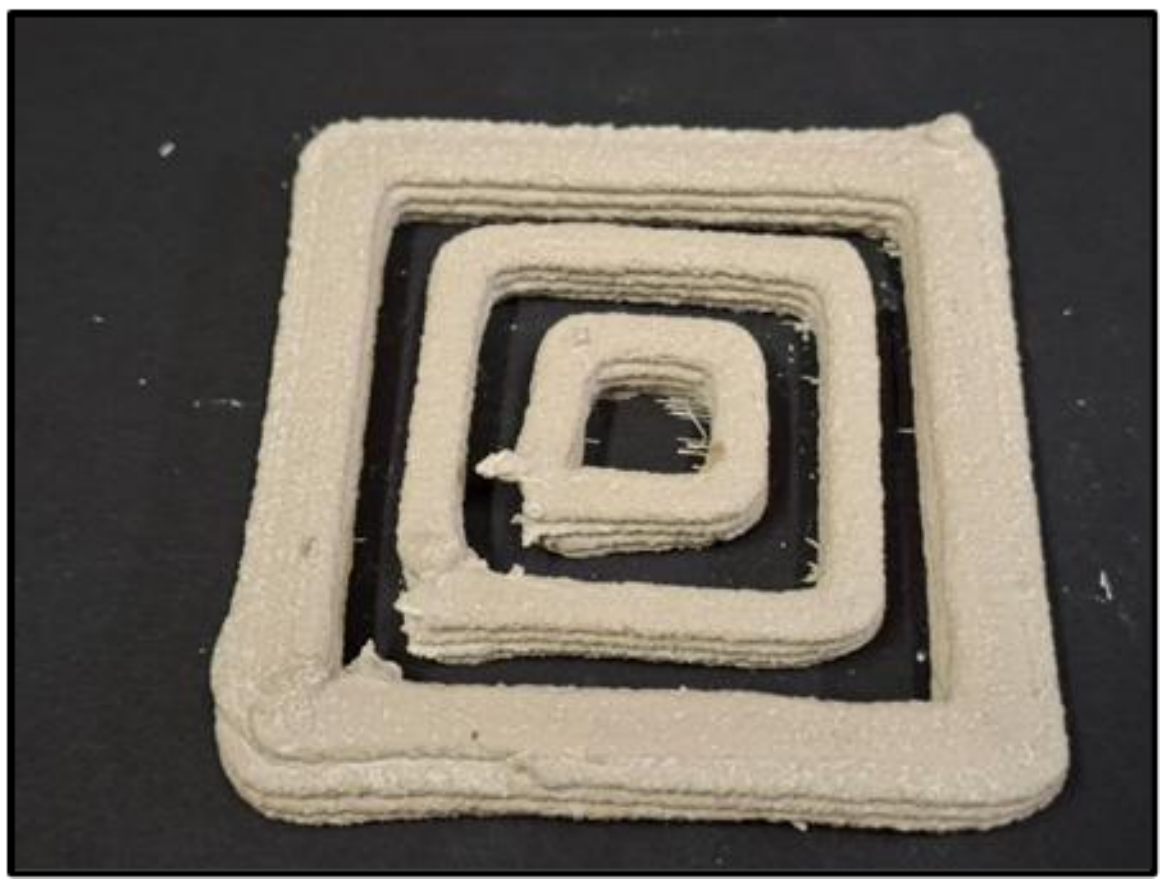

Figure S15. Left: pattern - triangles; density - 20%; fill angle - 90°; infill/perimeters overlap - 40%; seams - random. Right: pattern - concentric; density - 40%; fill angle - 90°; infill/perimeters overlap - 40%; seams - aligned.

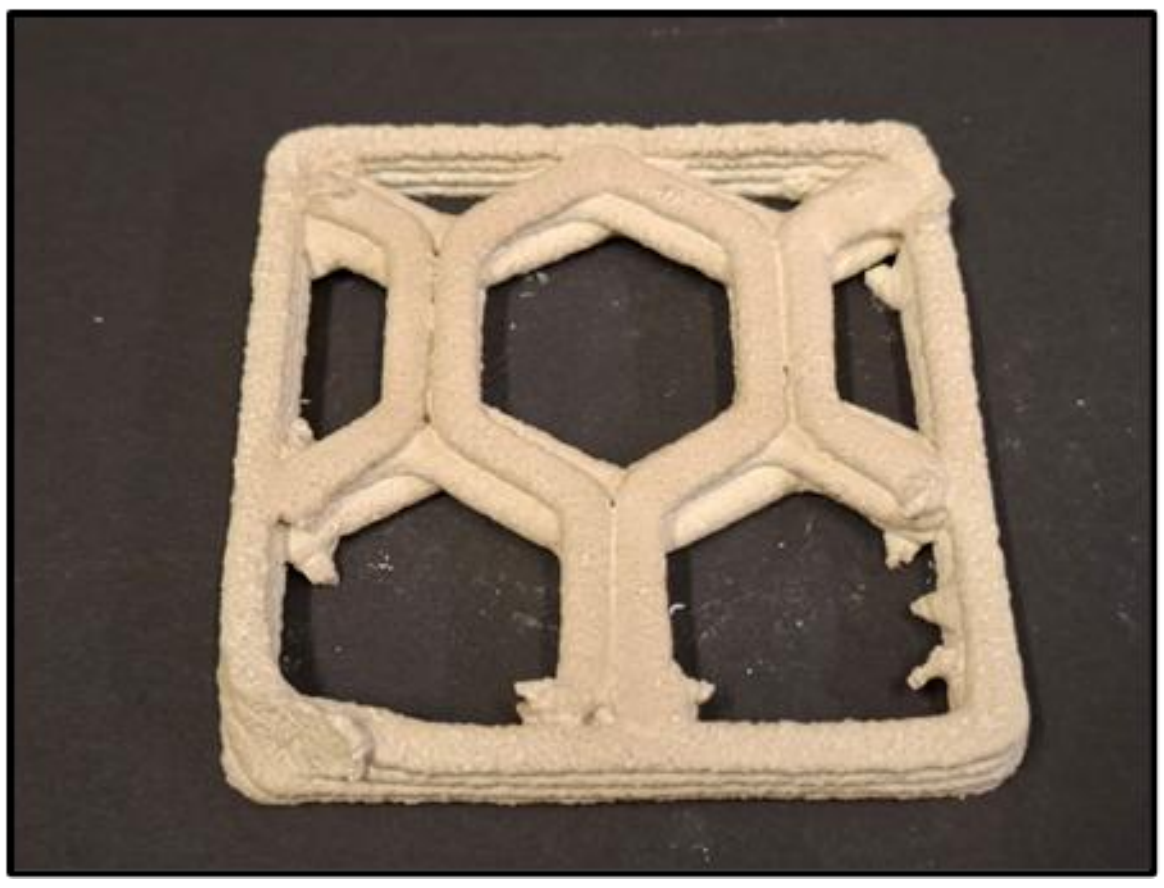
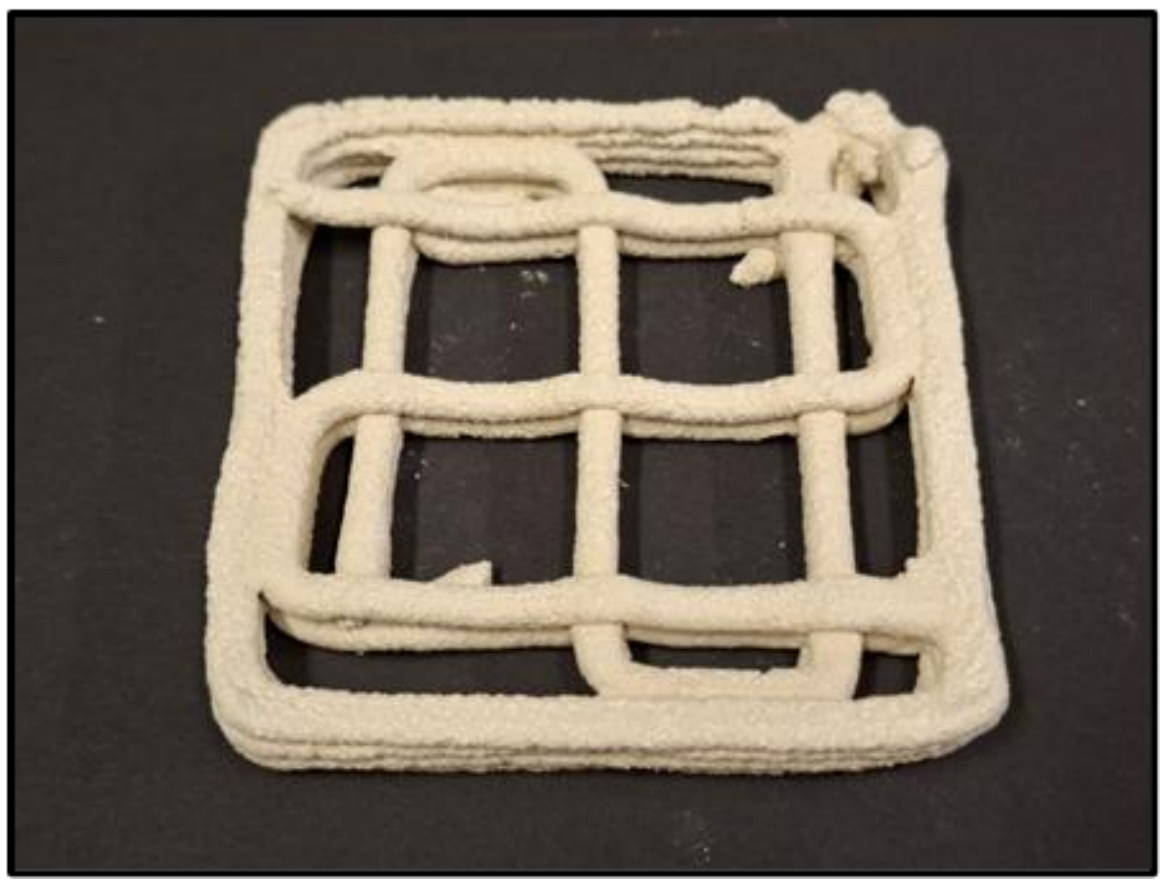

Figure S16. Left: pattern - honeycomb; density - 25%; fill angle - 90°; infill/perimeters overlap - 40%; seams - random. Right: pattern - rectilinear; density - 20%; fill angle - 90°; infill/perimeters overlap - 20%; seams - aligned.

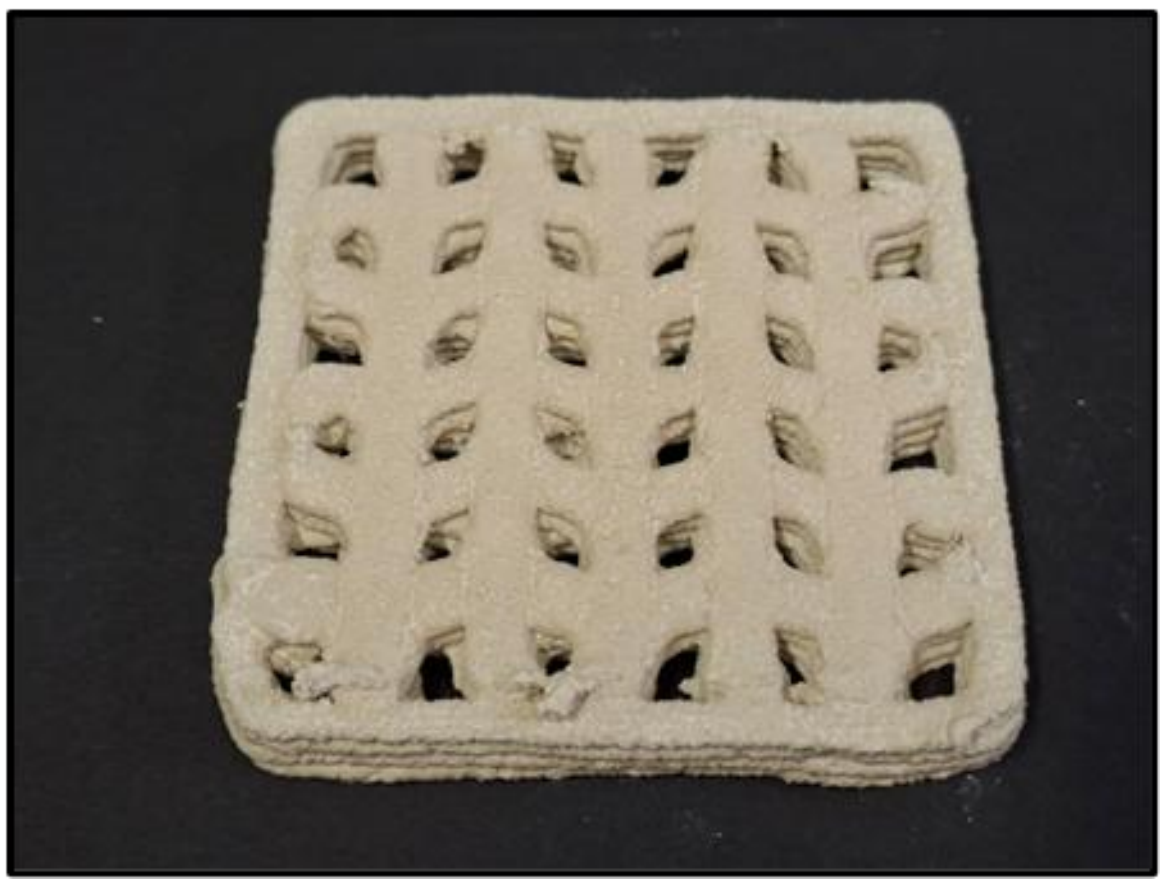

Figure S17. Pattern - grid; density - 80%; fill angle - 90°; infill/perimeters overlap - 40%; seams - random.

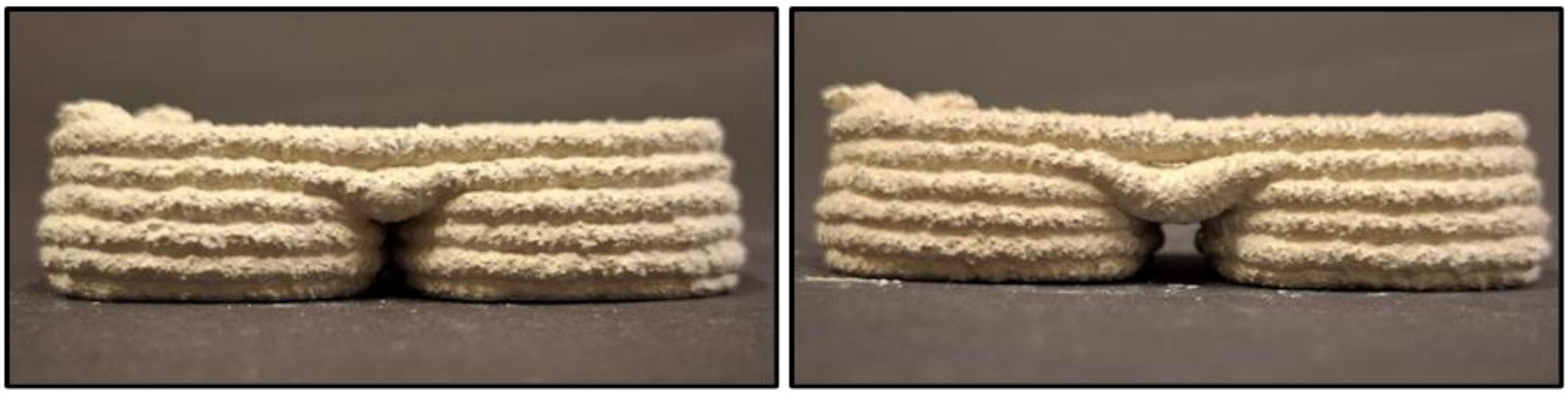

Figure S18. Left: bridge gap - 5 mm; infill/perimeters overlap - 40%; only bottom layers. Right: bridge gap - 10 mm; infill/perimeters overlap - 40%; only bottom layers.

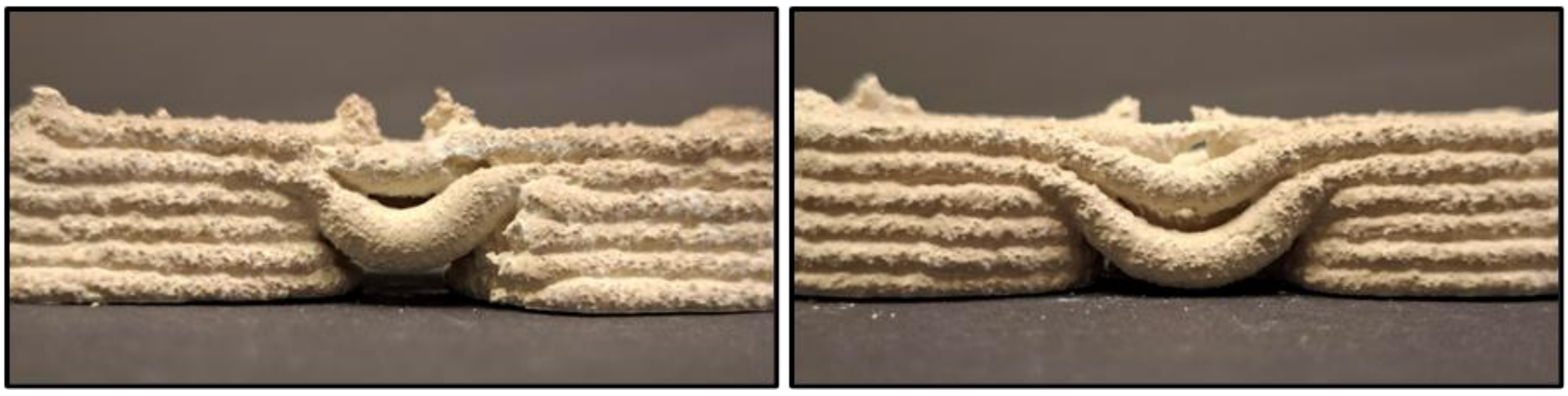

Figure S19. Left: bridge gap - 15 mm; infill/perimeters overlap - 40%; only bottom layers. Right: bridge gap - 20 mm; infill/perimeters overlap - 40%; only bottom layers.

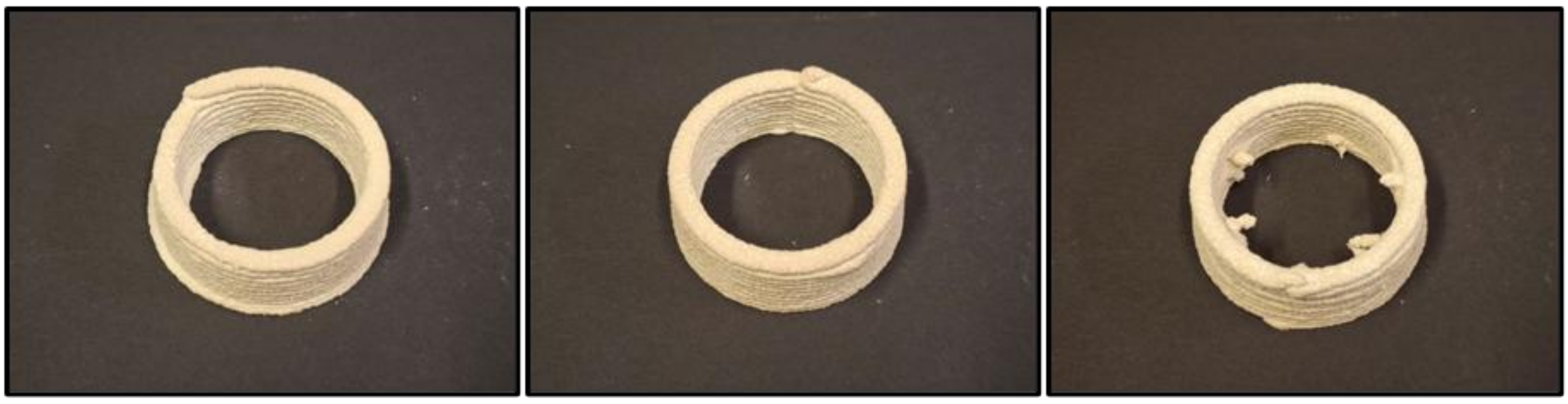

Figure S20. Left: spiral vase style perimeter. Middle: perimeter seams aligned. Right: perimeter seams random.

SI section 5 – Dog-bone tensile testing

Sample printing and curing

All samples were printed in sets of 2 and cured in 100% relative humidity and 21±1 °C environment for 3 days, after which the samples were removed from the print sheet.

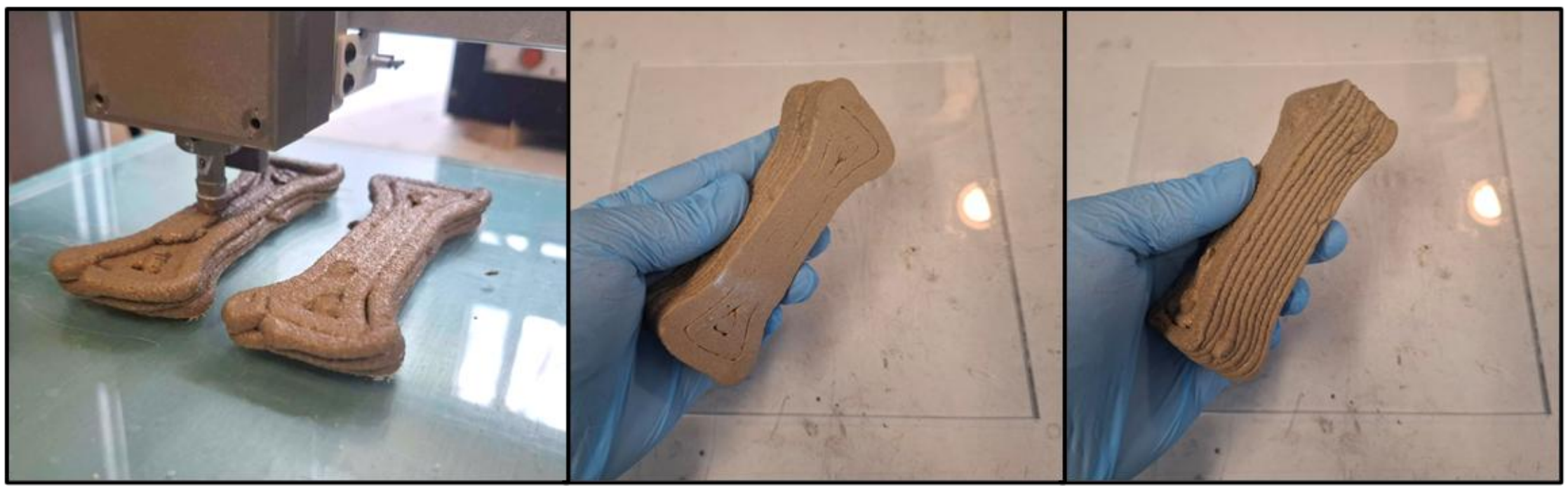

Figure S21. Dog-bone samples during printing and after removal from print sheet.

Before further curing, wet cutting was performed to ensure unform geometry and to remove print defects, if present. Printed samples were 10% thicker (in z-axis direction) than optimal for tensile testing. That design was intentional, as most of the print defects were confined to the first few printed layers, and were removed in wet cutting.

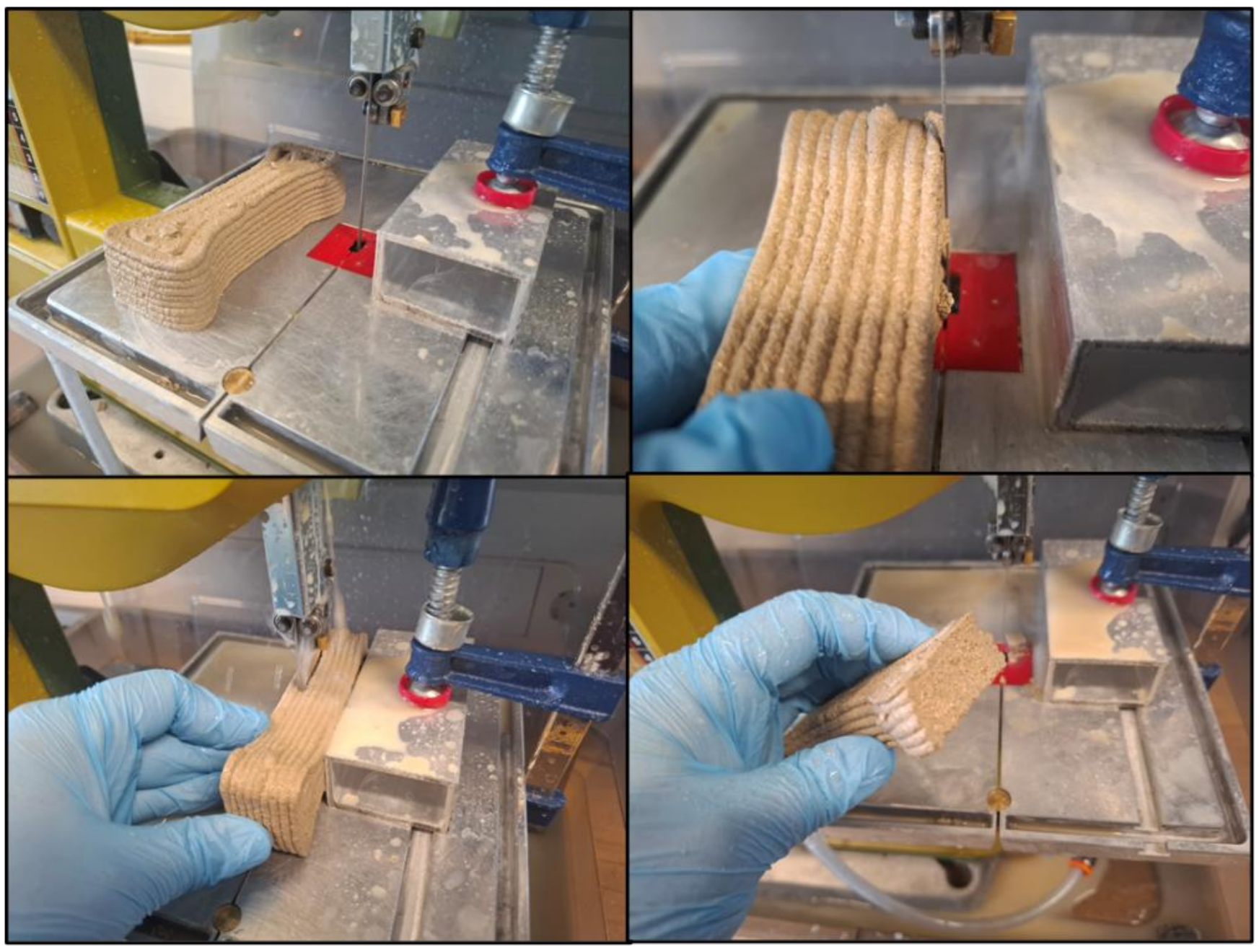

Figure S22. Wet cutting of dog-bone samples.

After cutting the samples were cleaned with compressed air to remove any loose debris, packed into film at 100% relative humidity and 21±1 °C and cured further, until 7 days of age, followed by drying and tensile testing.

Tensile testing

Tensile testing was performed utilising entirely friction gripping (Figure S23). Rubber padding was glued to the inner sides of aluminium plates to archive high degree of surface friction. The plates were tensioned by M4x50 mm bolts and nuts, allowing for a high degree of control over the compressive stresses within one clamp (two tensioned plates). The fixing points on each clamp allowed for two degrees of freedom, ensuring uniaxial tension along the specimen main longitudinal axis.

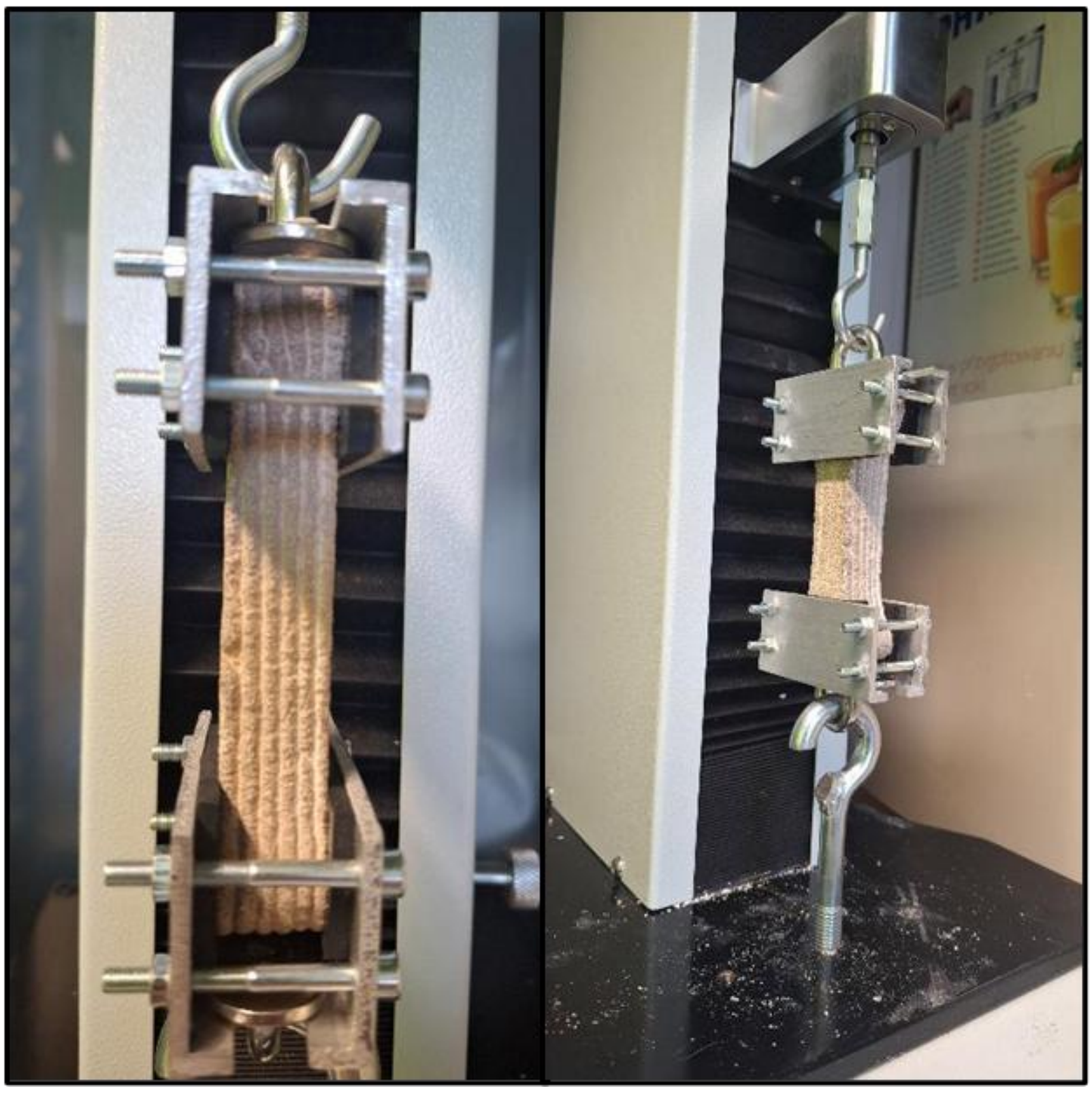

Figure S23. Tensile testing rig.

SI section 6 – Mix preparation

In the following detailed mix preparation methodology is provided. At first stage, alkaline reagent was premixed for 24 hours on magnetic stirrer. Reagents were added in the following order: NaOH, sodium silicate, milli-rho H_2O, Na-carboxymethyl cellulose (Na-CMC).

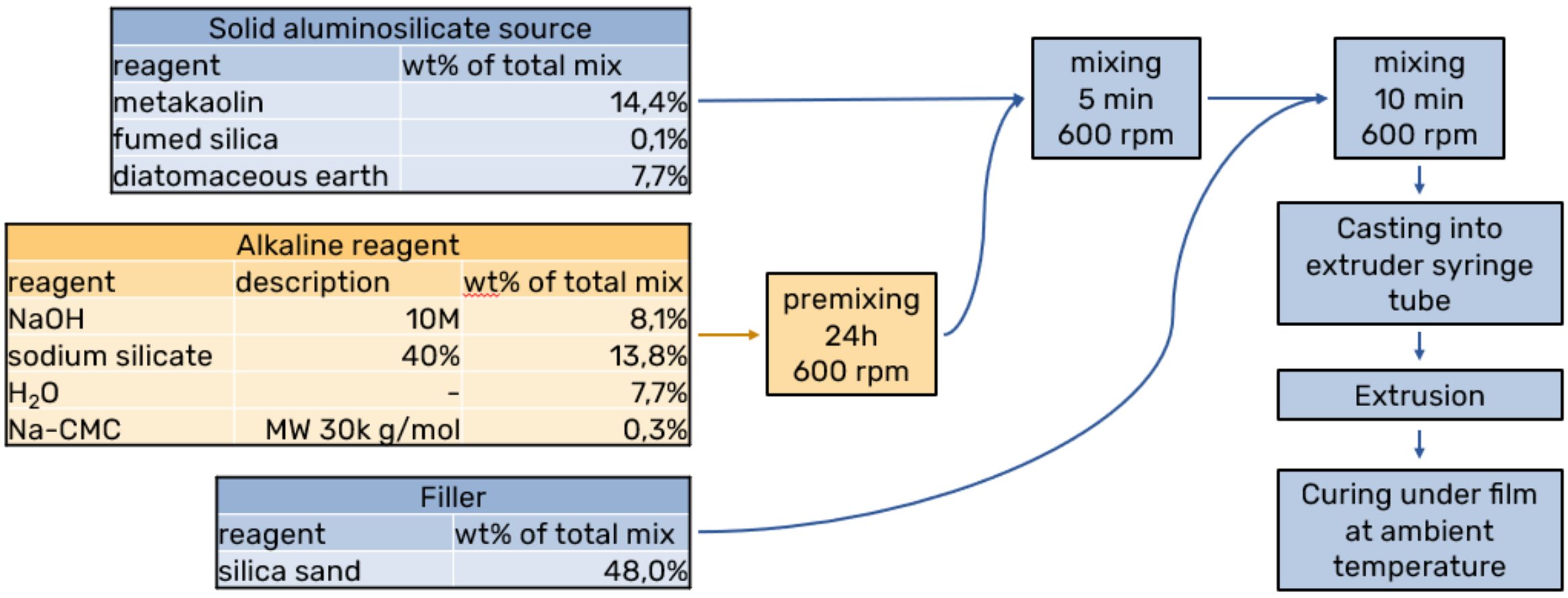

Figure S24. Mix preparation stage 1 – alkaline reagent premixing.

After 24 hours the alkaline reagent was transferred to stainless steel mixing container. Aluminosilicate reagents were then added at evenly spaced time intervals during 5 minutes one-by-one in the following order: metakaolin, diatomaceous earth, fumed silica.

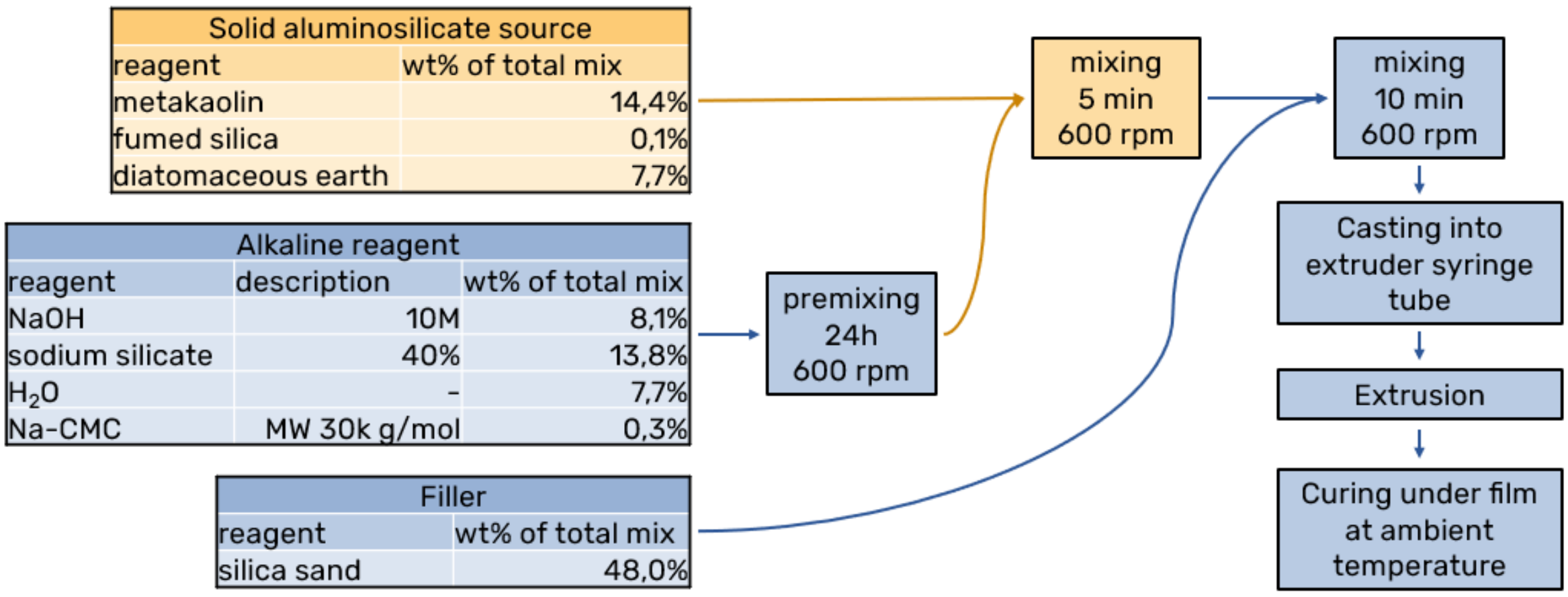

Figure S25. Mix preparation stage 2 – aluminosilicate reagent mixing.

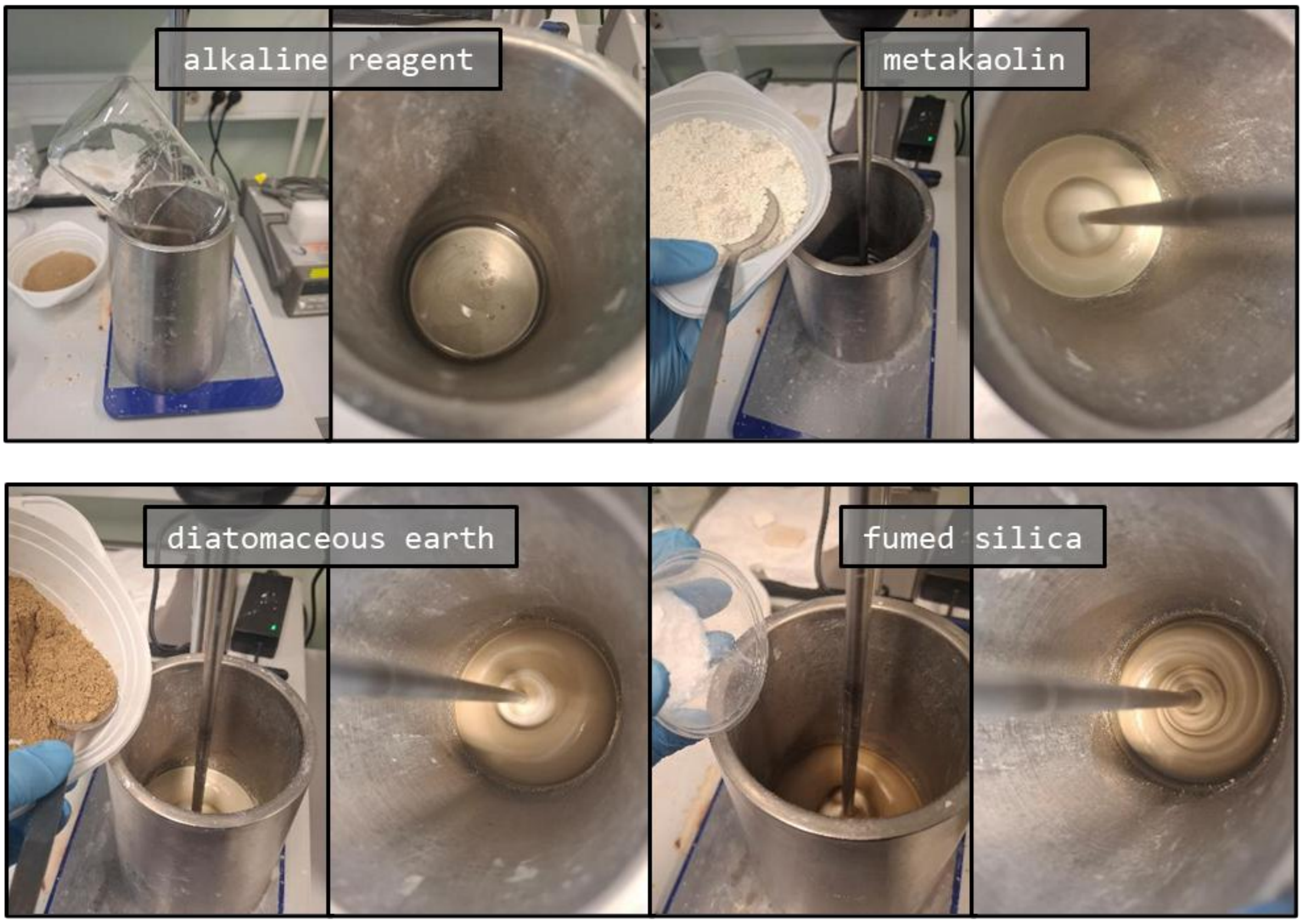

Figure S26. Photos depicting alkaline reagent (not premixing) and aluminosilicate reagent stage by stage mixing in the steel mixing container.

After 5 minutes of adding aluminosilicate reagents and mixing, filler material, i.e. sand, was added and mixed during 1 minute and then mixed for further 9 minutes.

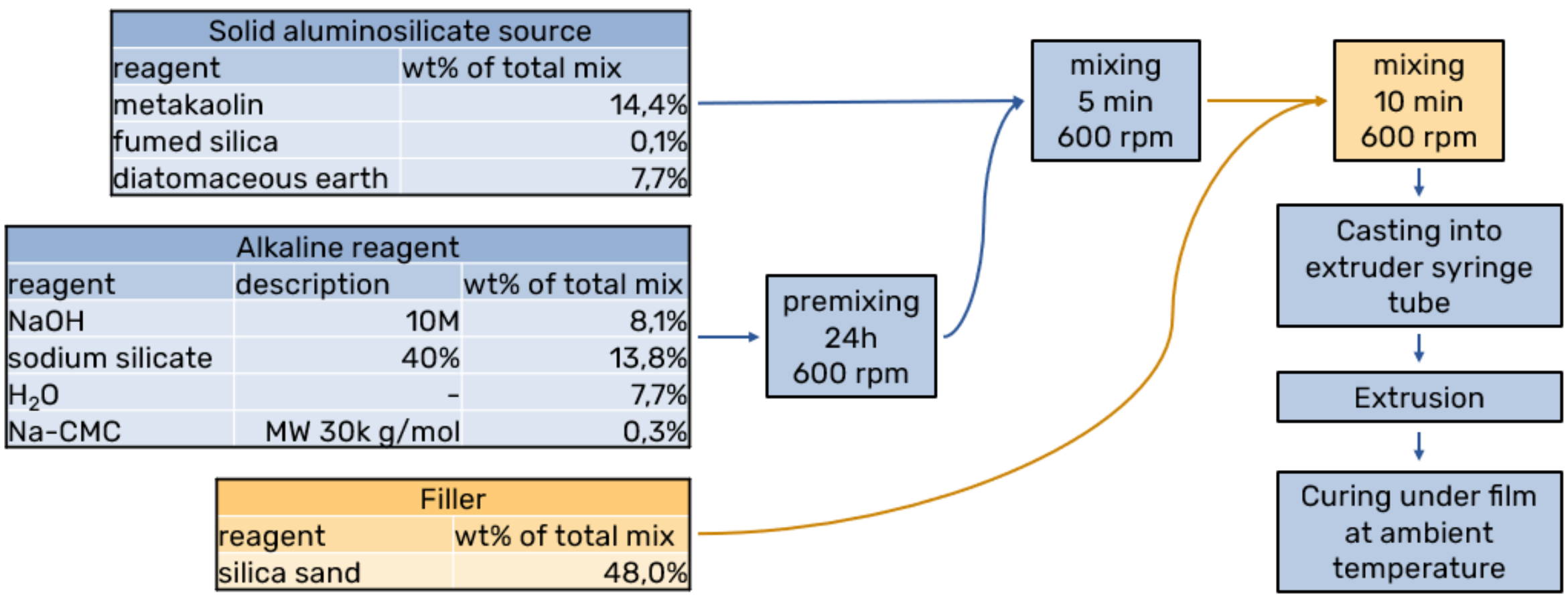

Figure S27. Mix preparation stage 3 – filler mixing.

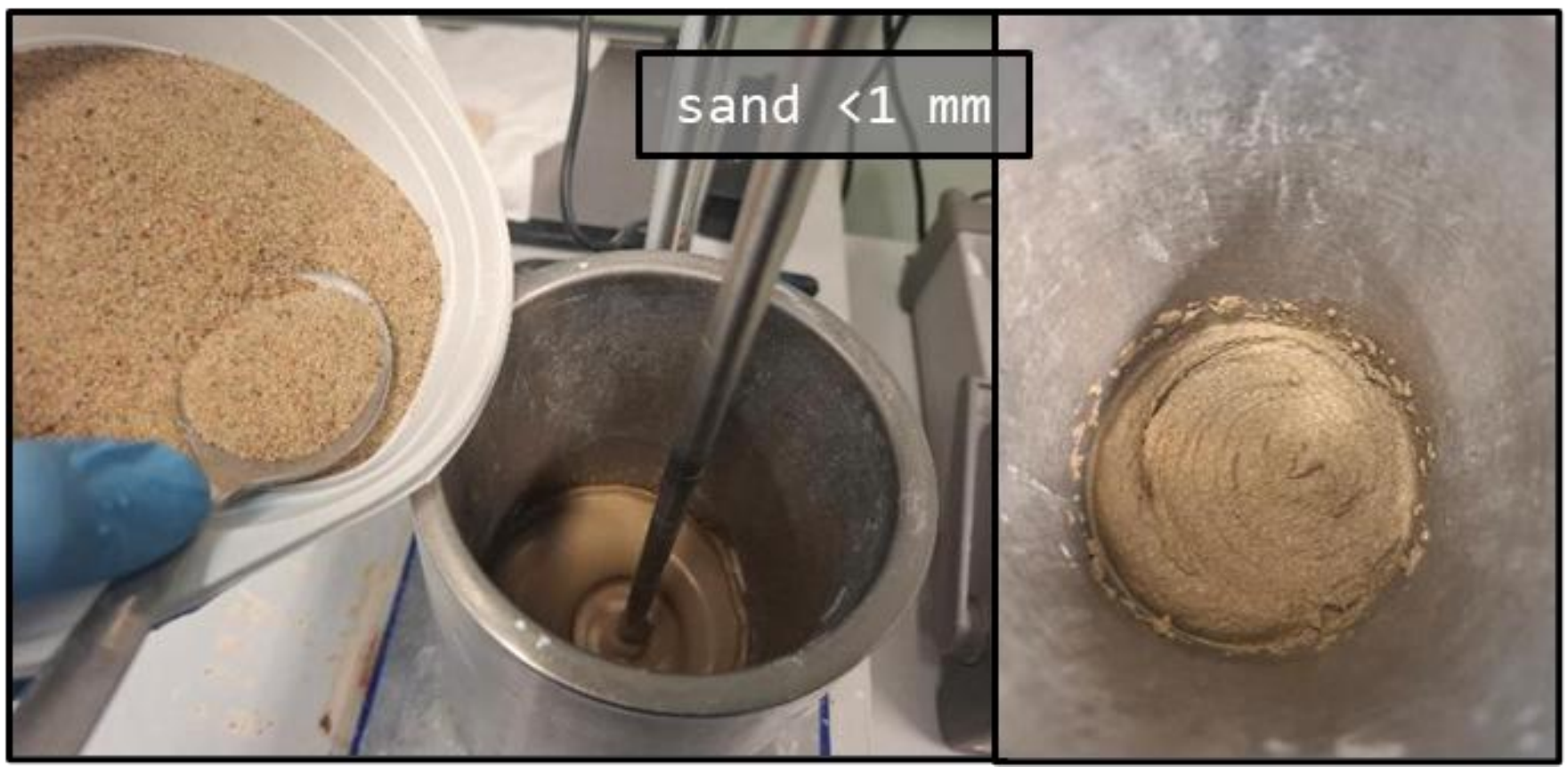

Figure S28. Photos depicting filler mixing in the steel mixing container.

Then the material was cast/shovelled into printer syringe container. Material was compacted mechanically with a stick or by manually shaking it. Vibration was not used and is not recommended (unless very well controlled), due to bleed effects.

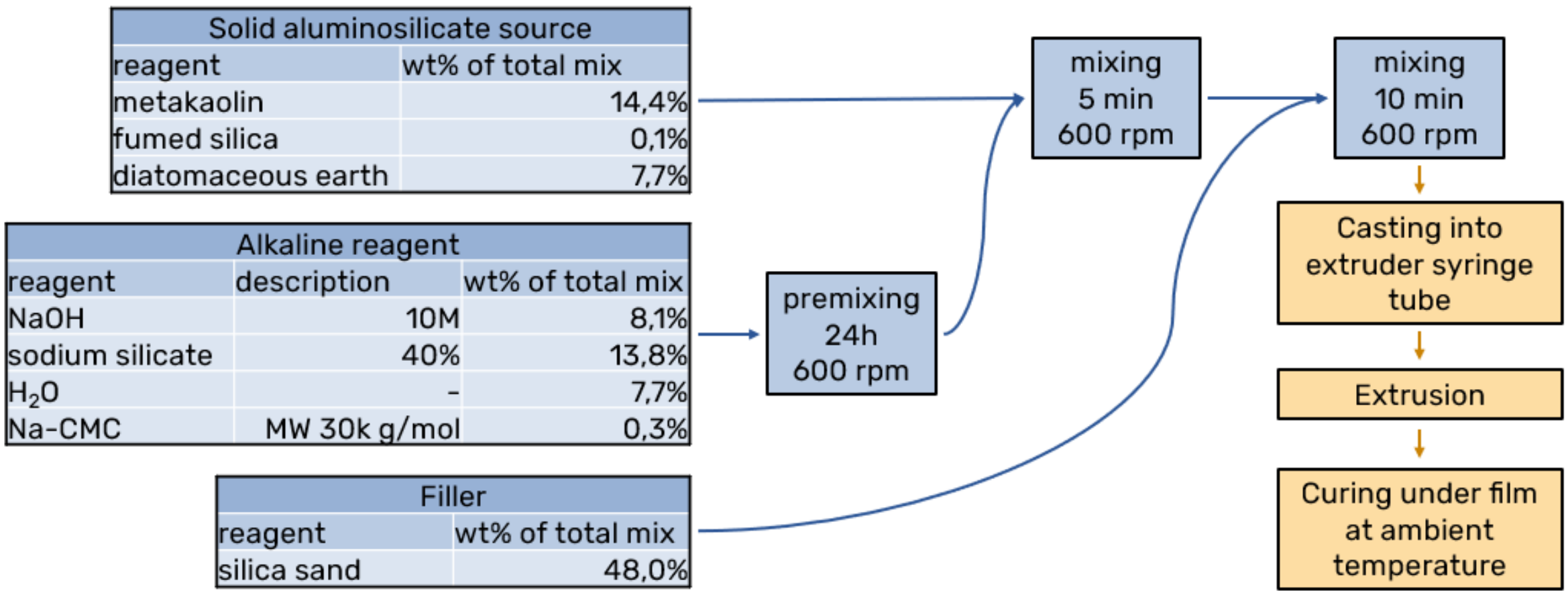

Figure S29. Mix preparation stage 4 – casting into the syring tube (and the following extrusion).

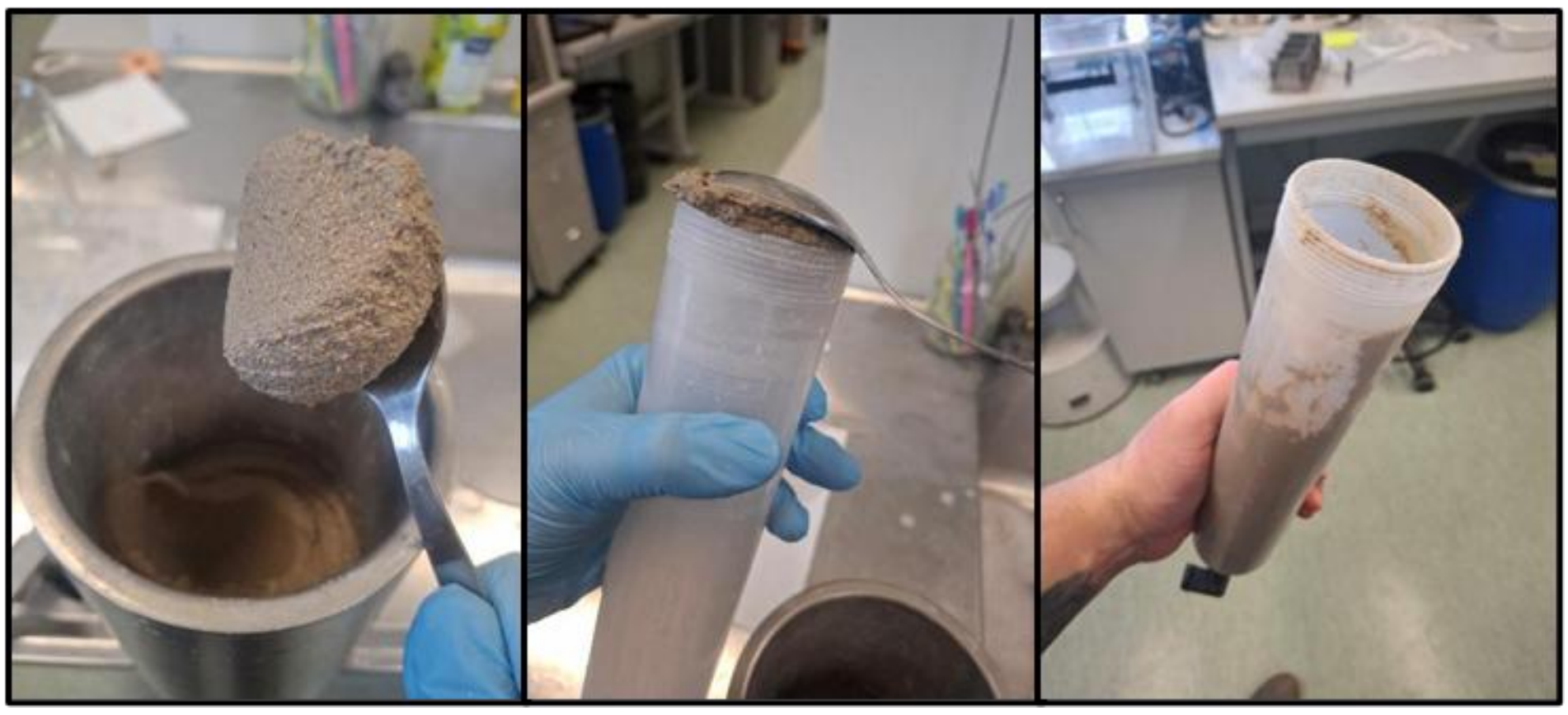

Figure S30. Photos depicting the material casting into the syringe tube.

SI section 7 – Chemical composition of raw materials

The chemical compositions (Table S2) of metakaolin, diatomaceous earth and sodium silicate (MR ≈ 2.9, aq.) were obtained from supplier datasheets. The remaining precursors were treated as 100% pure for the purposes of compositional calculations.

Table S2. Chemical compositions of raw materials.

<table>
<tr><th rowspan="2">Oxide equivalent</th><th colspan="3">Content (wt.%)</th></tr>
<tr><th>Metakaolin</th><th>Diatomaceous earth</th><th>Sodium silicate</th></tr>
<tr><td>SiO_2</td><td>52.3</td><td>85</td><td>29.6</td></tr>
<tr><td>Al_2O_3</td><td>45.2</td><td>5</td><td>-</td></tr>
<tr><td>Fe_2O_3</td><td>0.42</td><td>3</td><td>-</td></tr>
<tr><td>K_2O</td><td>0.15</td><td rowspan="9">7</td><td>-</td></tr>
<tr><td>TiO_2</td><td>1.74</td><td>-</td></tr>
<tr><td>Na_2O</td><td>0.22</td><td>10.4</td></tr>
<tr><td>CaO</td><td>0.04</td><td>-</td></tr>
<tr><td>MgO</td><td>0.04</td><td>-</td></tr>
<tr><td>P_2O_5</td><td>0.08</td><td>-</td></tr>
<tr><td>Other</td><td>-</td><td>-</td></tr>
<tr><td>H_2O</td><td>-</td><td>60.0</td></tr>
<tr><td>LOI</td><td>0.79</td><td>-</td></tr>
</table>
